# RNase Y-mediated regulation of the streptococcal pyrogenic exotoxin B

**DOI:** 10.1080/15476286.2018.1532253

**Published:** 2018-10-18

**Authors:** Laura Broglia, Solange Materne, Anne-Laure Lécrivain, Karin Hahnke, Anaïs Le Rhun, Emmanuelle Charpentier

**Affiliations:** a Max Planck Unit for the Science of Pathogens, Berlin, Germany; b Department of Regulation in Infection Biology, Max Planck Institute for Infection Biology, Berlin, Germany; c Institute for Biology, Humboldt University, Berlin, Germany; d Department of Regulation in Infection Biology, Helmholtz Centre for Infection Research, Braunschweig, Germany; e The Laboratory for Molecular Infection Medicine Sweden (MIMS), Umeå Centre for Microbial Research (UCMR), Department of Molecular Biology, Umeå University, Umeå, Sweden

**Keywords:** *Streptococcus pyogenes*, *speB*, RNase Y, virulence, 5′ untranslated region, transcriptional regulation

## Abstract

Endoribonuclease Y (RNase Y) is a crucial regulator of virulence in Gram-positive bacteria. In the human pathogen *Streptococcus pyogenes*, RNase Y is required for the expression of the major secreted virulence factor streptococcal pyrogenic exotoxin B (SpeB), but the mechanism involved in this regulation remains elusive. Here, we demonstrate that the 5′ untranslated region of *speB* mRNA is processed by several RNases including RNase Y. In particular, we identify two RNase Y cleavage sites located downstream of a guanosine (G) residue. To assess whether this nucleotide is required for RNase Y activity *in vivo*, we mutated it and demonstrate that the presence of this G residue is essential for the processing of the *speB* mRNA 5′ UTR by RNase Y. Although RNase Y directly targets and processes *speB*, we show that RNase Y-mediated regulation of *speB* expression occurs primarily at the transcriptional level and independently of the processing in the *speB* mRNA 5′ UTR. To conclude, we demonstrate for the first time that RNase Y processing of an mRNA target requires the presence of a G. We also provide new insights on the *speB* 5′ UTR and on the role of RNase Y in *speB* regulation.

## Introduction

Bacterial pathogens employ a plethora of mechanisms to tightly control the expression of their virulence factors. Among these mechanisms, RNA-mediated regulation provides bacteria with an efficient strategy to quickly respond to environmental stimuli [1,2]. Ribonucleases (RNases) play a critical role in RNA metabolism and have been largely associated with the regulation of factors involved in bacterial pathogenicity [3,4]. In particular, the single-stranded specific endoribonuclease Y (RNase Y) is crucial for the virulence of Gram-positive pathogens such as *Staphyloccocus aureus, Clostridium perfringens* and *Streptococcus pyogenes* [5–9]. In *S. aureus*, RNase Y stabilizes the mRNA coding for the two-component system SaeRS, which acts as a general regulator of virulence [8,10]. It also controls virulence gene expression indirectly, by affecting for example the production and stability of potential pathogenicity regulators such as the small RNAs (sRNAs) *rsaA* and *sau63* [8]. In *C. perfringens*, RNase Y regulates the expression of multiple virulence proteins. For example, upon binding of the sRNA VR-RNA, RNase Y processes the transcript coding for the collagenase toxin (ColA), resulting in increased stability of this transcript [9]. In *S. pyogenes*, RNase Y affects the expression of numerous virulence genes during the stationary growth phase [7]. However, the mechanisms by which RNase Y controls virulence expression in this bacterium are uncharacterized [7,11].

In some of the RNase Y targets described, the processing was shown to occur in proximity of RNA structures and in adenosine/uridine rich regions [10,12]. For instance, RNase Y cleaves the *yitJ* riboswitch and the *sae* mRNA operon upstream of double-stranded RNA structures, in *Bacillus subtilis* and *S. aureus* respectively [10,12]. In *S. aureus*, RNase Y processing events were found preferentially after a guanosine (G) [13]. Yet, direct evidence of the signals required for RNase Y to process RNAs in those bacteria is missing and nothing is known about the requirements for RNase Y processing in *S. pyogenes*.

*S. pyogenes* is a strict human pathogen that causes a broad range of diseases from mild to life-threatening infections [14]. In this bacterium, RNase Y affects the expression of a major virulence factor, the cysteine protease streptococcal pyogenic exotoxin B (SpeB) [7,11]. SpeB is the most abundant secreted protein from *S. pyogenes*. It degrades a variety of substrates, both from the bacteria and the host [15,16]. The degradation of streptococcal surface proteins and components of the host tissue by SpeB increases bacterial dissemination [15,16]. The production of SpeB is also particularly crucial in the development of necrotizing fasciitis [17,18] and acute post-streptococcal glomerulonephritis [19], two severe diseases associated to infections by *S. pyogenes*. SpeB is produced as an inactive zymogen, which undergoes maturation processes to become active. The biogenesis of the active SpeB protease is tightly regulated at both transcriptional and post-translational levels [20]. *speB* expression is controlled by various transcriptional regulators, including the well-characterized regulator of protease B (RopB) [21–23]. RopB binds at direct and inverted repeats located upstream of the *speB* promoter to induce *speB* expression [23,24]. The *speB* and *ropB* genes are adjacent on the chromosome and transcribed in opposite directions. A 940 nt-long intergenic region separates these two genes and contains numerous features [23]. It consists of two predicted open reading frames (ORFs) of unknown function [25] and also encodes the SpeB Inducing Peptide (SIP) involved in RopB-mediated regulation of *speB* expression [26]. It has recently been shown that a partial deletion of the *speB* 5′ UTR results in a global accumulation of mRNAs at the stationary phase of growth [27]. *speB* harbours a long 5′ UTR where two transcriptional start sites (TSSs), named P1 and P2, were originally predicted 697 nt and 137 nt upstream of the *speB* start codon, respectively [28]. Despite the important regulatory role of the intergenic region in *speB* expression, the annotation of this region is not consistent in the literature [21,23,26–29].

In a strain lacking RNase Y (∆*rny*), both *speB* mRNA and SpeB protein levels are highly downregulated [7,11]. Although previous reports proposed that RNase Y processes the *speB* transcript [11,30], this has never been validated. In addition, it has been hypothesized that several RNases target the *speB* mRNA 5′ UTR, but their identities and their effects on *speB* transcript abundance and stability are currently unknown [27]. Therefore, the molecular mechanism of RNase Y-mediated regulation of *speB* expression remains to be deciphered.

Here, we investigated the RNase Y-based regulation of *speB* expression and provide insight into the mechanism of RNase Y activity in *S. pyogenes*. We re-annotated *speB* TSSs and describe that at least two RNases, including RNase Y, process the 5′ UTR of *speB* mRNA. We show for the first time that a G residue is required for the processing of the *speB* transcript by RNase Y. Lastly, we demonstrate that RNase Y also activates the transcription of *speB*.

## Results

### Characterization and re-annotation of the *speB 5′* UTR

**Figure 1.**
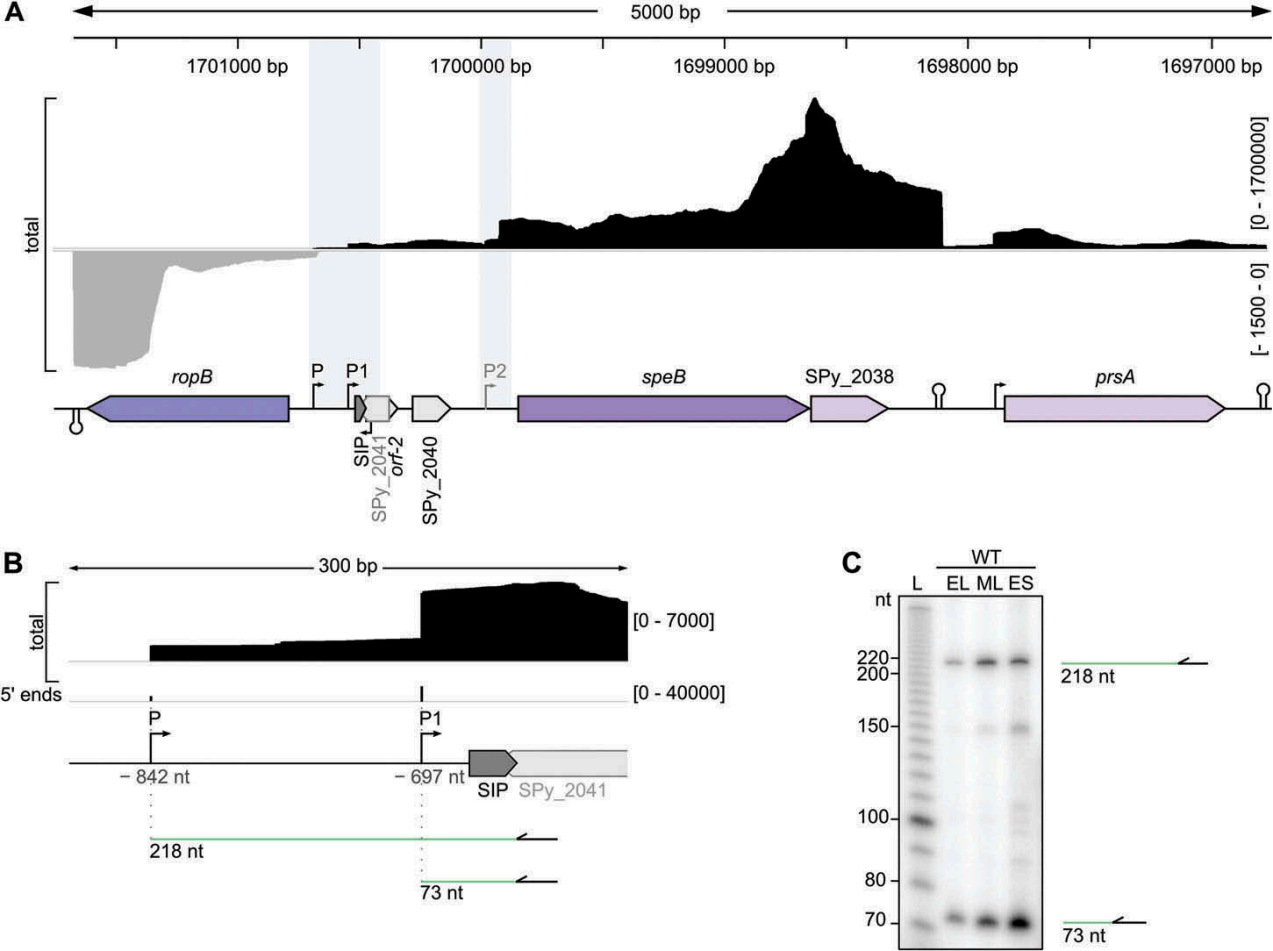
Characterization and re-annotation of *speB* 5′ UTR. (A) Expression profile of *speB* locus with surrounding genes (purple arrows) and putative open reading frames (ORFs, grey arrows) obtained by RNA sequencing analysis. Total coverages (black for positive strand, grey for negative strand) are indicated between square brackets. The genomic coordinates are shown and the putative promoters and terminators are indicated. *prsA* (encoding the foldase protein PrsA) is involved in the maturation of SpeB protease [28]. SPy_2038 was described to encode an inhibitor of SpeB protease activity [58]. *ropB* (encoding for the Regulator of protease B) is a transcriptional activator of *speB* expression [23]. The *speB*−*ropB* intergenic region contains several putative ORFs: SPy_2040, *orf-2*, the SpeB Inducing Peptide (SIP) and SPy_2041. P and P1 are the *speB* transcriptional start sites (TSSs); P2 depicts a 5′ end previously described as a *speB* TSS [23] and recently annotated as a processing site [27]. Features within the grey rectangles in the *speB* 5′ UTR are shown in more detail in Figs. 1B and 2–S2. (B) Expression profile of a portion of the *speB* 5′ UTR obtained by RNA sequencing analysis. Total and 5′ end coverages are indicated between square brackets. *speB* TSSs designated as P and P1 at positions –842 nt and –697 nt relative to the *speB* start codon, respectively, are shown with black bent arrows. A putative ORF (SPy_2041) and SpeB inducing peptide (SIP) are represented in grey. The green lines depict the length of cDNA products obtained by primer extension analyses and the primer used for the reaction is represented by the black arrows. (C) Validation of *speB* mRNA 5′ ends by primer extension analyses in the WT strain at early-logarithmic (EL), mid-logarithmic (ML) and early-stationary (ES) growth phases. The bands corresponding to the cDNA products, starting from the primer and terminating to the P or P1 5′ end, are indicated.

To characterize and identify additional features of the *speB* mRNA 5′ UTR, we performed total RNA sequencing of *S. pyogenes* M1GAS strain SF370 at early stationary phase of growth. Using these data, we annotated the 5′ boundaries of *speB* mRNA and identified two putative TSSs located 697 and 842 nt from the start codon of *speB* (Figs. 1A and S1A). We mapped the –10 boxes of P and P1 promoters by visual screening, and identified a putative –35 motif for P. Both P and P1 TSSs were validated by primer extension analyses at early-logarithmic (EL), mid-logarithmic (ML) and early-stationary (ES) growth phases (Fig. 1B,C). Previous studies show that the RopB binding sites are located upstream of P and P1 [23,26], indicating that RopB could promote *speB* expression from both promoters (Fig. S1A). In agreement with our data, P1 (–697 nt) was previously described as a *speB* TSS [23] and position P (–842 nt) was recently captured by 5′ rapid amplification of cDNA ends but not further characterized [27].

**Figure 2.**
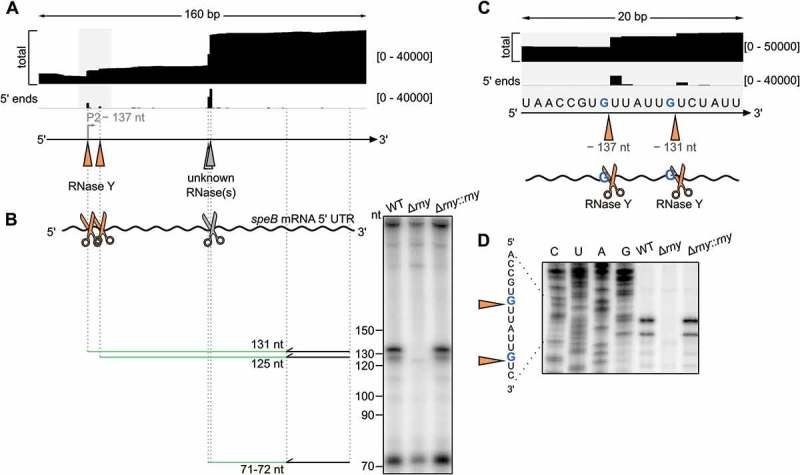
The *speB* mRNA 5′ UTR is targeted by ribonucleases. (A) RNA sequencing data and annotation of processing sites in the *speB* mRNA 5′ UTR. This region of interest is highlighted by a grey rectangle and shown in the context of the complete locus in Fig. 1A. The total and 5′ end coverages are indicated between square brackets. The positions corresponding to the cleavage sites by RNase Y and by unidentified RNase(s) are represented with orange and grey triangles, respectively. The bent grey arrow indicates a 5′ end previously described as a *speB* TSS (P2) [23] and re-annotated as a RNase Y processing site in this study. (B) Primer extension analyses of transcripts starting at P2 position in WT, *rny* (RNase Y) deletion mutant (∆*rny*) and chromosomal complemented *rny* deletion mutant (∆*rny::rny*) at early-stationary (ES) growth phase. The primer (black arrow) anneals to the *speB* 5′ UTR (curved black line) upstream of the processing by RNase Y (orange scissors) and by unidentified RNase(s) (grey scissor). The expected cDNA products (green lines) starting from the primer and ending to the cleavage sites are shown beside the corresponding bands. (C) Zoom on RNase Y cleavages confirmed by primer extension in the *speB* mRNA 5′ UTR (orange triangles and scissors) at positions –131 nt and –137 nt relative to the *speB* start codon and mapped after a guanosine (G). (D) Primer extension analyses showing RNase Y processing sites of *speB* mRNA 5′ UTR in WT, ∆*rny* and ∆*rny::rny* strains at ES growth phase. A sequencing ladder was used in order to annotate the RNase Y processing sites at the exact nucleotide position (orange triangles). The lanes C, U, A and G indicate the RNA sequence in the region of interest.

The *ropB-speB* intergenic region harbors several poorly characterized small ORFs. Originally, two putative ORFs were annotated: SPy_2040 (upstream of *speB*) and SPy_2041 (upstream of *ropB*) [25] (Fig. S1B). A different organization of the intergenic region has recently been proposed: three ORFs were annotated, SPy_2040 (named *orf-3*) SIP and *orf-2* [26]. In accordance with this annotation, RNA sequencing reveals that SPy_2041 is not transcribed in our experimental conditions (Fig. S1B). Indeed, a single TSS (P*_ropB_*) was mapped downstream of the SPy_2041 start codon, at position –369 nt relative to the *ropB* start codon.

### The *speB* mRNA 5′ UTR is targeted by ribonucleases

An additional TSS (P2) located 137 nt upstream of the *speB* start codon was previously proposed, however this promoter was not sufficient to initiate transcription of a reporter gene in the absence of P1 [23]. Recently, it has been hypothesized that this 5′ end, located in the 5′ UTR, results from processing of the *speB* mRNA [27]. We also observed a transcript 5′ end at the original P2 position by RNA sequencing, but failed to identify putative – 10 and – 35 boxes. To investigate the origin of this 5′ end, we performed primer extension analyses on this region of *speB* using total RNA from wild type (WT) and strains deleted from various endoRNases and exoRNases (Figs. 2A and S2A). A cDNA fragment corresponding to a transcript starting at P2 (–137 nt) was detected in all the strains tested except in ∆*rny*, indicating that RNase Y is involved in producing this 5′ end (Figs. 2B and S2A). Therefore, we re-annotated P2 as a processing site of RNase Y. A second RNase Y processing event was also retrieved in the *speB* 5′ UTR, 6 nt downstream of P2, and further validated (Fig. 2A,B). Interestingly, both processing events were located after a G (Fig. 2C), which was confirmed by primer extension analyses (Fig. 2D). These two transcript 5′ ends were both present in the complemented *rny* deletion mutant (∆*rny::rny*) (Fig. 2B,D).

We also detected an additional transcript 5′ end located 77–78 nt upstream of the *speB* start codon by RNA sequencing and primer extension (Figs. 2A and S2). A search for –10 and – 35 motifs located upstream of this 5′ end was unsuccessful. In a previous study, reporter fusions containing up to 623 bp upstream of this 5′ end were not expressed, indicating the lack of promoter activity in this region [23]. Therefore, we hypothesized that this 5′ end resulted from the processing of *speB* mRNA 5′ UTR. We could not pinpoint the RNase(s) responsible for this event as the processing site was detected in all RNase deletion mutant strains analyzed (Fig. S2A). This processing is probably carried out by RNases that were not included in this study, for instance RNases J1 or J2, which are essential for *S. pyogenes* survival [31]. We also cannot exclude that more than one RNase is capable of cleaving at this specific position (*i.e*. when one RNase is deleted, the processing is carried out by alternative RNase(s)), a phenomenon defined as functional redundancy.

### Characterization of RNase Y processing sites in the *speB* mRNA 5′ UTR

**Figure 3.**
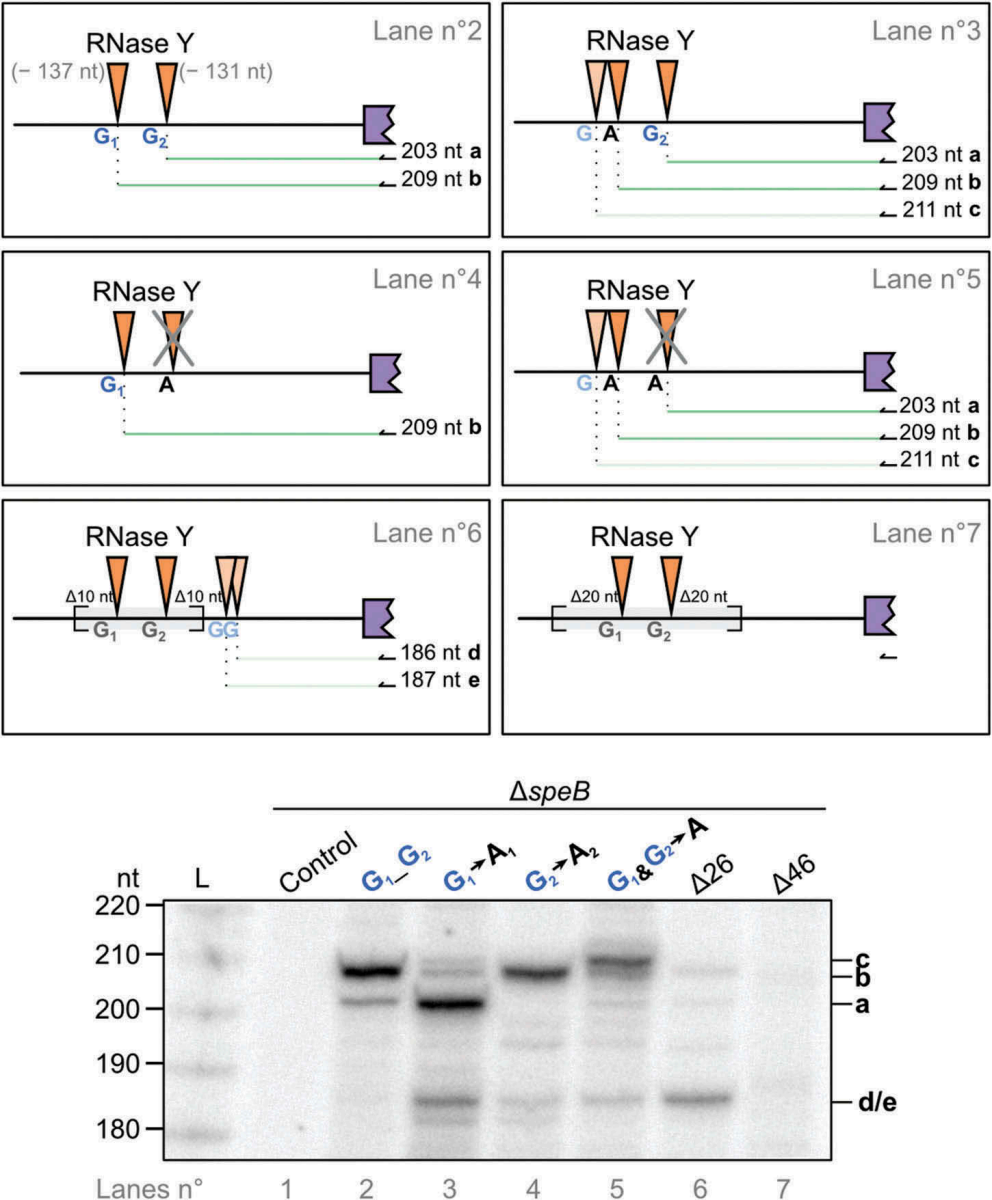
RNase Y requires a guanosine to process the 5′ UTR of *speB* mRNA. Characterization of *speB* mRNA processing by a mutational analysis of RNase Y cleavage sites. Primer extensions were performed at early-stationary growth phase in a *speB* deletion mutant (∆*speB*) containing different plasmids (top panel) expressing *speB* (including the 5′ UTR) under the control of a constitutive promoter. The primer (black arrow) anneals to the region corresponding to the beginning of the *speB* ORF. An empty vector was used as a control (Lane n°1). The cDNA products are depicted with green lines (a, b, c and d/e). G-to-A substitutions upstream of the RNase Y processing sites at positions –137 nt (G_1_) and –131 nt (G_2_) were done as indicated (lanes n° 2, 3, 4, 5). Plasmids used in lanes 6 (∆26) and 7 (∆46) harbor a deletion of 10 nt and 20 nt both upstream and downstream of RNase Y cleavages, respectively. Dark orange triangles represent RNase Y processing sites in the 5′ UTR of *speB* mRNA. Light orange triangles indicate putative alternative RNase Y processing sites.

It has previously been described that processing of transcripts by RNase Y occurs in 58% of the cases downstream of a G in *S. aureus* [13], but whether the presence of this nucleotide is essential for RNase Y activity was never investigated. To further examine the requirements for *speB* 5′ UTR processing, we ectopically expressed *speB* 5′ UTR and ORF under the control of a constitutive promoter (P*_gyrA_*) in a *speB* deletion mutant (∆*speB*). The two Gs located upstream of the RNase Y processing sites at positions –137 nt (G_1_) and –131 nt (G_2_) were independently or simultaneously substituted by adenosine (A) (Fig. 3, top panels). In addition, we deleted 26 nt (∆26) or 46 nt (∆46) encompassing the RNase Y cleavage sites (Fig. 3, top panels). The effect of these mutations on RNase Y processing was examined by primer extension analyses (Fig. 3, bottom panel). While the substitution of G_1_ by A_1_ strongly reduced RNase Y processing (Fig. 3; lanes 3 and 5), that of G_2_ by A_2_ fully inhibited it (Fig. 3; lanes 4 and 5). When G_1_ was substituted by A_1_, an alternative cleavage site was detected a few nucleotides upstream of the original RNase Y processing site (Fig. 3; lanes 3 and 5). Interestingly, this alternative processing was also located after a G, 2 nt upstream of the initial RNase Y processing. Additionally, when 10 nt upstream and downstream of the processing site of RNase Y were removed, a substitute processing site in the *speB* mRNA 5′ UTR was also detected (Fig. 3; lane 6). We analyzed the *speB* 5′ UTR sequence and found two Gs (positions –115 nt and –116 nt relative to the *speB* start codon) possibly corresponding to those alternative processing events (Fig. 3; lane 6) likely generated by RNase Y. Finally, a deletion of 20 nt both upstream and downstream of the RNase Y processing sites completely abrogated the cleavages (Fig. 3; lane 7). Thus, we prove here for the first time that a G located just upstream of a RNase Y cleavage site is essential for the processing by this enzyme, in *S. pyogenes* and for the *speB* mRNA 5′ UTR.

To assess the structural context around RNase Y cleavages, we predicted the secondary structure of *speB* mRNA 5′ UTR neighboring the first RNase Y cleavage site (100 nt upstream and downstream) (Fig. S3A). The RNase Y processing events were located in a poorly structured region, coherent with the fact that RNase Y cleaves single-stranded RNA (Fig. S3A). We observed a decrease in the minimum free energy (∆G), indicating the presence of a secondary structure approximately 40 nt downstream of the RNase Y cleavage sites (Fig. S3A,B). This result is in contrast to what was recently shown in *S. aureus*, where RNase Y recognizes a secondary structure element located 6 nt downstream of the processing event [10]. A model in which RNase Y processing occurs through a 6 nt (from the structure) ruler and cut mechanism was proposed. A similar scenario was previously observed in *B. subtilis*, where RNase Y processes the *yitJ* riboswitch, 6 nt upstream of the aptamer structure [12]. In *speB* mRNA, RNase Y processing events were 6 nt apart, however as mentioned above in a poorly structured region, and thus it would be unlikely that the ruler and cut mechanism by RNase Y is applicable for *speB* mRNA in *S. pyogenes*.

## speB *mRNA abundance is downregulated in* ∆rny

**Figure 4.**
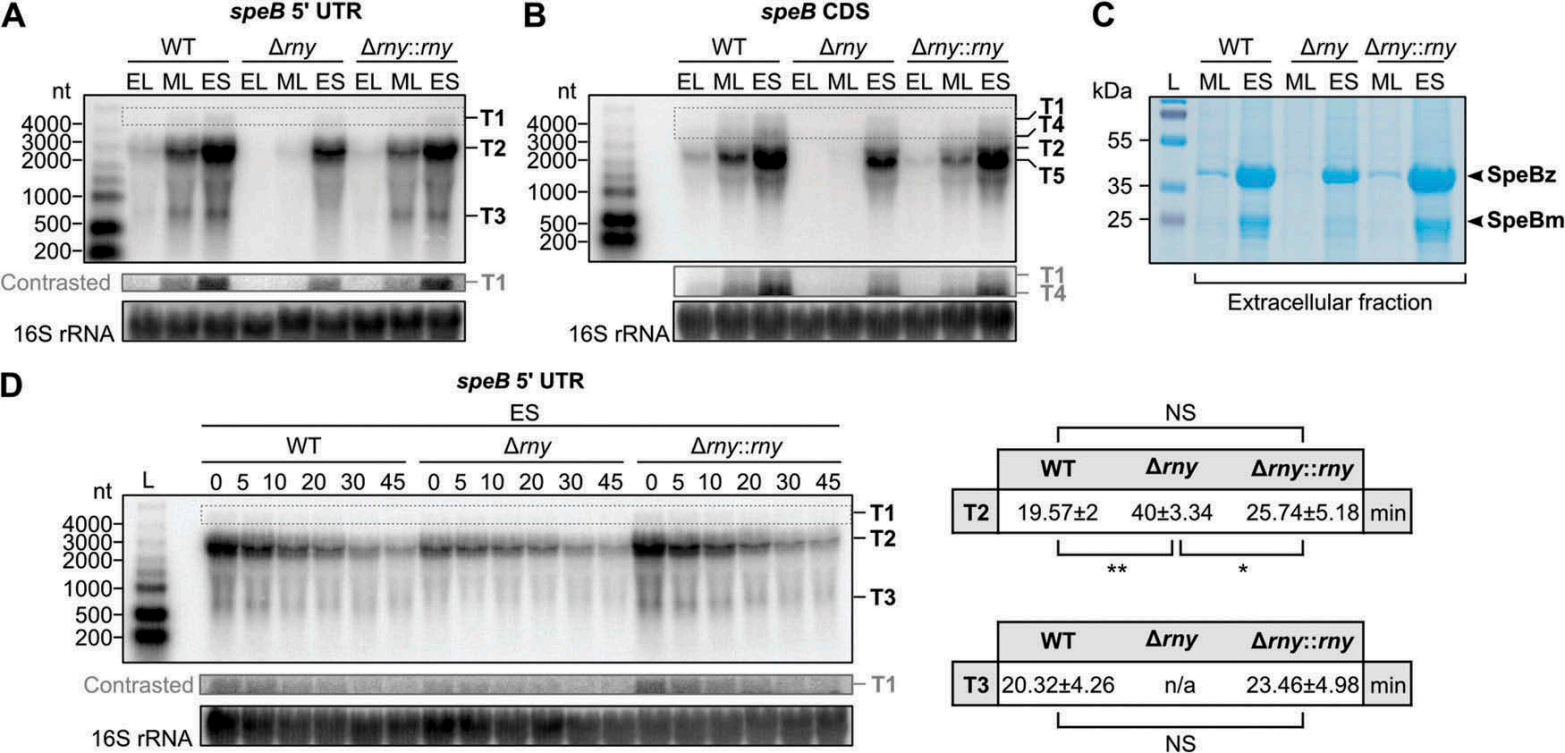
RNase Y regulates *speB* expression. (A–B) *speB* transcript abundance assessed by Northern blot analysis performed in WT, *rny* (RNase Y) deletion mutant (Δ*rny*) and chromosomal complemented *rny* deletion mutant (∆*rny::rny*) grown until early-logarithmic (EL), mid-logarithmic (ML) and early-stationary (ES) growth phases. The primers used target *speB* mRNA 5′ UTR (A) or *speB* CDS (B), see also Fig. S4. (C) Analysis of SpeB protein levels in WT, Δ*rny* and ∆*rny::rny*. Extracellular protein fractions from ML and ES growth phases, resolved by SDS-PAGE and visualized by Coomassie blue staining. SpeB zymogen (SpeBz, 40 kDa) and SpeB mature form (SpeBm, 28 kDa) are indicated. (D) Study of *speB* transcript stability by rifampicin assay at ES growth phase (left panel). The minutes after stopping transcription upon addition of the antibiotic are indicated. The transcript isoforms detected (T1, T2, T3, T4 and T5) and the primer used for the hybridization reaction are shown in Fig. S4. 16S rRNA was used as a loading control. The calculated half-lives in minutes of T2 and T3 isoforms are shown in the right panel. The half-life of T1 isoforms could not be determined with accuracy due to the intense background of the blots. The values shown indicate the average ± standard deviation. The half-lives were compared using a t-test, single and double asterisks denote a p-value inferior of 0.05 and 0.01, respectively. NS indicates no significant difference.

In order to study the effect of RNase Y processing in *speB* regulation, we analyzed the temporal pattern of *speB* expression by Northern blot analysis and the production of SpeB protease by SDS-PAGE (Fig. 4). *speB* expression was previously shown to be growth phase dependent and affected by nutritional and environmental conditions [20]. In accordance with those data, we observed an increase of *speB* mRNA abundance from EL to ES growth phases (Fig. 4A). Three distinct groups of *speB* isoforms were detected by using a primer targeting *speB* 5′ UTR. Co-transcription of *speB* together with the genes located immediately downstream (SPy_2038 and *prsA2*) led to the production of ~4 kb transcripts (T1) (Figs. 4A and S4). The most abundant transcripts, T2, arose from *speB* promoters and ended at the SPy_2038 terminator (Figs. 4A and S4). Smaller isoforms (T3) which could derive from the cleavages of *speB* mRNA by RNase Y and/or by unknown RNase(s) were also detected (Figs. 4A and S4). However, since T3 isoforms were not visible in ∆*rny*, they probably resulted mostly from RNase Y processing. The processed isoforms produced by unidentified RNase(s) were not detected (Fig. 4A). It is possible that the upstream transcripts derived from cleavage by the unidentified RNase(s) were rapidly degraded in contrast to that produced by RNase Y. Using a primer binding to the *speB* coding DNA sequence (CDS), two additional isoforms (T4 and T5), downstream of the processing events in the *speB* mRNA 5′ UTR, were observed (Figs. 4B and S4). The most abundant isoforms were T5 rather than the primary transcripts (T2) (Figs. 4B and S4). We hypothesize that the T5 isoforms observed in ∆*rny* were generated by the unidentified RNase(s). The *speB* transcript abundance was strongly reduced at both ML and ES stationary growth phases in the ∆*rny* mutant and restored in ∆*rny::rny* (Fig. 4A). In agreement with this result, the level of extracellular SpeB protease, decreased in ∆*rny*, was rescued in ∆*rny::rny* (Fig. 4C).

To assess if the decrease of *speB* abundance in ∆*rny* was due to a reduction of *speB* transcript stability, we performed rifampicin assays (Fig. 4D). We did not observe any destabilization of *speB* transcripts in the absence of RNase Y. On the contrary, the half-life of the *speB* isoforms T2 was higher in ∆*rny* (40 min ± 3.34) compared to WT (19.57 min ± 2) (Fig. 4D), confirming that RNase Y cleaved this transcript.

### RNase Y regulates *speB* expression at the transcriptional level

**Figure 5.**
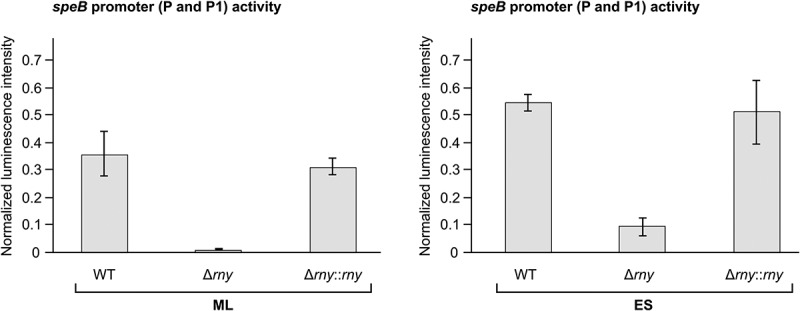
RNase Y regulates *speB* expression at the transcriptional level. The *speB* promoter activity was examined by luminescence assays performed in WT, *rny* (RNase Y) deletion mutant (∆*rny*) and chromosomal complemented *rny* deletion mutant (∆*rny::rny*), containing the luciferase fusion plasmids (P23-*ffluc* (pEC2248) and P*speB-ffluc* (pEC2173), see also Fig. S5A) at mid-logarithmic (ML, left panel) and early-stationary (ES, right panel) growth phases. Values indicate the luminescence intensity of the samples relative to the plasmid control (P23-*ffluc*), normalized to the OD_620_ _nm_. Mean and standard (error bars) deviations were calculated from three independent experiments, each with technical triplicates.

As *speB* transcript stability was not decreased in ∆*rny*, we postulated that *speB* downregulation in this strain could be due to indirect transcriptional effects. To test this hypothesis, the expression of a reporter gene (firefly luciferase) under the control of the *speB* promoters was tested in WT, ∆*rny* and ∆*rny::rny* (Figs. 5 and S5A). The luminescence signal in ∆*rny* was really low, indicating that the *speB* promoters were less active in this strain (Fig. 5). In ∆*rny::rny*, the activity of the *speB* promoters was restored to the WT level. To exclude possible autoregulatory effects in these conditions, the *speB* promoter activity was also assessed in the ∆*speB* mutant. Our data show that the luminescence signals were comparable in WT and ∆*speB* (Fig. S5B).

Although RNase Y cleaves *speB* mRNA, we showed that the decrease of *speB* levels in ∆*rny* is not a direct consequence of this processing. Rather, RNase Y is indirectly involved in the activation of *speB* promoters. Taken together, our data indicate that, in *S. pyogenes* SF370 strain, RNase Y mainly modulates *speB* expression at the transcriptional level through an unknown intermediate factor.

*speB* expression is controlled by several transcriptional regulators, but their regulation by RNase Y is poorly described (Supplementary Table II). We tested, in our conditions, the stability of the *covRS* transcript, encoding the CovRS two-component system, a repressor of *speB* transcription. The *covRS* transcript stability was highly increased in ∆*rny* compared to the WT strain (Fig. S6), as previously suggested [29]. Therefore, CovRS could be the missing link between RNase Y and *speB*.

## Discussion

In this study, we investigated the role of RNases, in particular RNase Y, in the regulation of the virulence factor SpeB in *S. pyogenes*. In an effort to provide coherence for future studies – the annotation of the *speB* 5′ UTR has been inconsistent in previous works [23,26–28] –, we first determined *speB* transcript boundaries. We demonstrated that RNase Y and so far unidentified RNase(s) process the 5′ UTR of *speB* mRNA.

Yet, the consequences of these processing events remain unknown and we propose different scenarios. Processing of *speB* mRNA 5′ UTR could lead to the production of a mature form of *speB* mRNA that would affect the translation of *speB* mRNA. For instance, in *S. aureus*, the processing of the 5′ UTR of *cpsA* mRNA (encoding a major cold shock protein) by RNase III promotes ribosome binding and translation of *cpsA* mRNA [32]. In addition, the truncation of the *speB* 5′ UTR was shown to induce an early onset of *speB* expression and an increase of bulk mRNA stability [27]. Therefore, it is also possible that the processed *speB* 5′ UTR functions as a *trans*-acting regulatory molecule. Indeed, RNA sequencing analyses previously revealed the presence of a putative sRNA (Spy_sRNA1699993) located in the *speB* 5′ UTR [33] (depicted in Fig. S7). Interestingly, the sRNA 5′ end corresponds to the RNase Y cleavage site annotated at –137 nt from the *speB* start codon, therefore the biogenesis of this sRNA could result from RNase Y processing of *speB* mRNA 5′ UTR. Remarkably, the cleavage site performed by the unidentified RNase lies between the sRNA 5′ and 3′ ends (Fig. S7) suggesting that the sRNA is functional. Indeed, RNase processing represents a common mechanism to generate active sRNAs [34–36]. Alternatively, the entire 5′ UTR could act as a *trans*-acting RNA similar to the 5′ UTR of *irvA* mRNA of *Streptococcus mutans*, encoding a transcriptional repressor, that interacts with *gbpC* mRNA and prevents its cleavage by RNase J2 [37]. Another possibility is that the *speB* mRNA 5′ UTR is targeted by a sRNA regulating *speB* expression, which in turn would trigger the processing of the targeted transcript. This is the case in *C. perfringens*, where VR-RNA binds to the 5′ UTR of *colA* mRNA, resulting in the processing of this transcript by RNase Y and an increase in its stability [9,38].

Notably, both RNase Y processing sites of *speB* mRNA 5′ UTR were mapped after a G. A preference for a G upstream of RNase Y processing sites was previously described in *S. aureus* [13]. Here, we used *speB* mRNA as a model target to determine the importance of this residue for RNase Y activity. We show for the first time that a G is required for *in vivo* processing of *speB* mRNA by RNase Y. A similar sequence preference has been recently described for RNase E, the functional equivalent of RNase Y in Gram-negative bacteria. Global mapping of RNase E processing events in *Salmonella enterica* revealed the preference for a uridine (U), 2 nt downstream of the cleavage site [34]. Mutation of this nucleotide strongly reduced the processing of the mRNA targets by RNase E, both *in vitro* and *in vivo* [34]. Since structural data showed that RNase E interacts with an RNA substrate, 2 nt downstream of the cleavage site, the authors proposed a model in which the U at this position induces a conformational change in the catalytic site, which favors the processing. A similar explanation might be applicable to RNase Y, however the crystal structure of this enzyme is not available and therefore the mechanism used to recognize the G is challenging to investigate.

We observed that the amount of transcripts starting at the first RNase Y cleavage site (–137 nt) was higher than the transcripts starting at the second cleavage site (–131 nt) (Fig. 2A,B). A possible explanation is that the latter are degraded faster than the former. An alternative hypothesis is that RNase Y cleaves at these two positions with a different efficiency. In this scenario, other stimuli sensed by RNase Y could influence the preferred location for the processing.

Different determinants affecting RNase Y activity, such as secondary structures of the RNA target and RNase Y interacting proteins, have independently been investigated in other Gram-positive bacteria. Although previous studies showed that RNase Y is sensitive to secondary structures located downstream of the processing events [10,12], a recent transcriptomic analysis in *B. subtilis* did not identify any consensus motif (neither sequence nor RNA structure) in proximity of RNase Y cleavages [39]. Our mutational study indicates that the sequence requirement (*i.e*. G upstream the cleavage site) is essential for correct processing. However, we cannot exclude that secondary structures play a role in concert with the G residue in determining the RNase Y cleavage site location in *S. pyogenes*. In the structural prediction of *speB* mRNA 5′ UTR, we did not detect a double-stranded structure, 6 nt downstream of the cleavage sites that would resemble the structure described for *S. aureus* and *B. subtilis*. Therefore, if the structure is also involved in determining RNase Y target specificity, the mechanism is probably different from the one proposed in the aforementioned bacteria.

It has recently been shown that the processing by RNase Y of specific mRNA targets in *B. subtilis* depends on the so-called Y-complex, composed of RNase Y and interacting proteins [39,40]. Overall, the exact requirements for RNase Y activity *in vivo* still need to be determined, and current knowledge indicates that this enzyme may exhibit various characteristics in different bacteria. Our characterization of RNase Y processing of *speB* mRNA 5′ UTR provides new insights into RNase Y determinants required for processing in *S. pyogenes* that could facilitate the study of other RNase Y targets. Indeed, introducing point mutations in the G residue of other RNase Y substrates might serve as a useful tool to investigate those transcripts without deleting RNase Y and without disrupting any putative complex formed by RNase Y and potential interacting proteins.

We observed that RNase Y had an effect on the stability of the primary transcript (T2), as it was cleaved by this RNase. In a previous study, only two *speB* transcripts (long and short) were identified by Northern blot analyses. The longer transcript, which was stabilized in ∆*rny* compared to WT [11], could correspond to the isoforms here named T2. The authors proposed that by cleaving *speB* mRNA, RNase Y yields a shorter *speB* transcript, more stable than its precursor [11]. However, the origin of this processed isoform remains unclear, and we hypothesize that it might derive from the processing by unidentified RNase(s) observed in our study.

We demonstrated that the decrease of *speB* mRNA abundance in ∆*rny* was not due to a reduction of *speB* mRNA stability (Fig. 4C). Instead, our data underpin a model in which RNase Y controls *speB* expression indirectly via an unidentified transcriptional regulator. A possible candidate that was proposed to be involved in RNase Y control of *speB* expression is RopB, an activator of *speB* transcription [7]. Indeed, ectopic expression of *ropB* in ∆*rny*, restored the production of SpeB to the WT level. However, it is likely that the downregulation of *speB* mRNA in ∆*rny* is not due to RopB activity since the stability of the *ropB* transcript is highly increased in ∆*rny* [27,29], and this would result instead in increased *speB* expression. We cannot conclude that *ropB* mRNA levels are the same in our settings, since different strains and growth conditions were used in our study. It is also possible that more than one transcriptional factor contributes to the RNase Y-mediated regulation of *speB*. Of note, SIP, which activates *speB* expression through RopB, was co-transcribed with *speB* and its expression was downregulated in ∆*rny*. Consequently, it is possible that a negative feedback loop involving SIP is established, explaining the strong reduction of *speB* mRNA level in ∆*rny*.

Overall, the mechanisms by which RNases regulate virulence gene expression in *S. pyogenes* are yet to be fully understood. For instance, a group of transcripts encoding virulence proteins (*e.g*. Streptolysin S and Streptodornase D) are highly stable during stationary growth phase as their degradation – by RNases J1/J2 and PNPase – is delayed compared to other mRNAs [41,42]. It was proposed that the differences in RNA decay initiation of these two groups of mRNAs rely on features (*e.g*. secondary structures) in the mRNA 5′ UTRs [42]. However, other unidentified requirements, apart from the 5′ UTR, are also needed to discriminate the different sets of mRNAs to allow differential stability of virulence-related mRNAs [31]. RNase Y is involved in the regulation of virulence in *S. pyogenes*, but to date no regulatory mechanisms have been elucidated. Here, we established that the predominant mechanism of RNase Y-based regulation of *speB* expression is at the level of transcription. However, we cannot exclude the possibility that RNase Y processing events of *speB* transcript exert a more significant regulation under different conditions – for example, in response to specific environmental stimuli.

In the present study, we have addressed the complexity of RNase Y regulation of a major virulence factor in *S. pyogenes*. Our data raise further important questions regarding the exact role, either direct or indirect, of RNase Y in the modulation of virulence in this bacterium, and may pave the way for further studies on the relevance of RNA processing as a regulatory mechanism of pathogenicity factor expression.

## Materials and methods

### Bacterial culture conditions

Bacterial strains are listed in Supplementary Table I. *S. pyogenes* SF370 (M1 GAS) was grown in THY medium (Todd Hewitt Broth) supplemented with 0.2% yeast extract (Servabacter®) without shaking at 37°C, with 5% CO_2_. For growth on solid medium, TSA blood agar (Trypticase^TM^ Soy Agar, Becton Dickinson) supplemented with 3% defibrinated sheep blood (Oxoid) was used. *Escherichia coli* was grown as described in [43]. When required, antibiotics were added to the medium at the following final concentrations: 3 μg/ml erythromycin and 300 μg/ml kanamycin for *S. pyogenes*; 300 μg/ml erythromycin and 50 μg/ml kanamycin for *E. coli*.

### Construction of gene deletion mutants

Chromosomal deletions of *rnhB, mrnC yhaM* (SPy_0267), *pnpA, rnr* and *speB* in *S. pyogenes* were performed using the Cre-Lox recombination system [44] as described in [43] with the following modifications. Briefly, regions upstream and downstream of *rnhB, mrnC, yhaM, pnpA, rnr* and *speB* were amplified by PCR from WT genomic DNA using oligos listed in Supplementary Table I. The last 156 nt of the *speB* ORF were not removed in order to keep the ribosome binding site (RBS) of the downstream gene (SPy_2038). These upstream and downstream regions were ligated to the lox71-*ermAM/B*-lox66 and cloned in suicide vectors for *S. pyogenes* (pSEVA141 for *yhaM* and *speB*; pUC19 for *rnhB, mrnC* and *pnpA*; pJET1.2 for *rnr*) (plasmids in Supplementary Table I). Prior to transformation in *S. pyogenes*, the plasmids were linearized in the *ampR* gene with SacII (for pSEVA141) or with PvuI (for pUC19 and PJET1.2). Insertion of the lox71-*ermAM/B*-lox66 cassette was checked by PCR and DNA sequencing (Microsynth, Switzerland). The *ermAM/B* cassette was then removed from ∆*rnhB*, ∆*mrnC, ∆pnpA, ∆rnr* and *∆speB* deletion mutants by expressing the Cre recombinase. The integrity of the generated strains was checked by PCR and DNA sequencing (Microsynth, Switzerland).

### Construction of chromosomal complemented ∆*rny* strain

The *S. pyogenes* ∆*rny* strain was complemented on the chromosome. A fragment including the *rny* gene together with the upstream region and the TT3 transcriptional terminator [45] was generated by PCR with OLEC3584/OLEC3579. The downstream region of *rny* and the lox71-*ermAM/B*-lox66 cassette were amplified by PCR with OLEC3480/OLEC3572 and OLEC2000/OLEC3585, respectively. The PCR products obtained were ligated and cloned in a suicide vector for *S. pyogenes* (pRS426 ATCC® 77107™) by applying the homologous recombination system of *Saccharomyces cerevisiae* S228C [46]. *S. cerevisiae* cells were grown in YPD medium (Yeast extract Peptone Dextrose) at 30°C until OD_620_ _nm_ of 0.6 and washed with water. Following an additional wash with SORB buffer (100 mM lithium acetate; 10 mM Tris-HCl pH 8; 1 mM EDTA pH 8; 1M sorbitol), the cells were resuspended in SORB buffer containing boiled salmon sperm (1.43 mg/ml). The competent cells obtained (50 µl) were incubated in PEG-buffer (100 mM lithium acetate; 10 mM Tris-HCl pH 8; 1 mM EDTA pH 8; 40% PEG3350) for 30 min with the PCR fragments (100 ng each) and plasmid pRS426 was linearized with BamHI/KpnI. *S. cerevisiae* was transformed by heat shock of 15 min at 42°C. Positive clones (growing on plates without uracil) were selected, pooled and the plasmids were extracted with the QIAprep® Spin Miniprep Kit following the manufacturer’s instructions. The lysis step was modified as follows: the cells were mixed with zirconium/glass beads (Ø 0.1 mm, Roth) and vortexed for 15 min. The heterogeneous plasmid preparation was introduced by transformation in *E. coli* TOP10 to obtain isolated plasmids carrying the correct insert. The plasmid was linearized with PvuI (cleaving in the *ampR* cassette) and introduced by transformation in electrocompetent *S. pyogenes*.

### Bacterial transformation

*E. coli* cells were transformed using a standard heat-shock protocol described in [47]. *S. pyogenes* competent cells for the deletion of *rnhB, mrnC, yhaM* (SPy_0267), *pnpA, rnr* and *speB* were prepared as in [43]. For transformation of *S. pyogenes* (WT or deletion mutants) with plasmids, competent cells were generated according to the procedure shown in [48] with some modifications. The electroporation was performed with a pulse of 1.8 kV, 400 Ω, 25 μF.

### Construction of plasmids for *speB* ectopic expression

The *speB* 5′ UTR and coding sequence were amplified from WT genomic DNA using primers OLEC7970/7971 and ligated to a *gyrA* promoter from *Streptococcus agalactiae* (P*_gyrA_* amplified from pEC455 using OLEC7968/7969) by PCR-mediated ligation with OLEC7968/OLEC7971. The resulting fragment was cloned in pEC85 digested with XbaI-EcoRI to generate plasmid pEC2146, which was used as a template to construct the variant plasmids with mutated *speB* 5′ UTR. The mutations were introduced with the two-stage PCR methodology [49], with primers listed in Supplementary Table I.

### RNA extraction

Total RNAs from *S. pyogenes* were prepared from cultures harvested at early-logarithmic (EL; OD_620nm_ = 0.1), mid-logarithmic (ML; OD_620nm_ = 0.25) and early-stationary (ES; OD_620nm_ = 0.4) growth phases. The samples were mixed with 1:1 ice-cold acetone/ethanol and the RNAs were precipitated using isopropanol (Sigma) following extraction with TRIzol (Life Technologies)/chloroform (Sigma). RNA concentrations were measured using NanoDrop (Thermo Scientific) and RNA integrity was determined by agarose gel electrophoresis.

### Rifampicin assay

Rifampicin (Sigma-Aldrich) was dissolved in methanol and added to the cultures at ES growth phase at a final concentration of 250 μg/ml. 25 ml of cultures were harvested immediately (0 min) and at defined time points (5, 10, 20, 30, 45 min) after the addition of rifampicin.

### Northern blot analysis

Total RNA (15 μg) was separated on a 1% agarose gel (1X MOPS (20 mM MOPS free acid, 5 mM sodium acetate, 1 mM EDTA, pH 7.0), 6.6% formaldehyde), in 1X MOPS buffer containing 0.7% formaldehyde. The RNAs were transferred onto a Nylon Hybond N+ membrane (GE Healthcare). The transfer was carried out on a capillary system overnight at room temperature in 20X SSC buffer. The cross-linking of RNAs on the 6X SSC-rinsed membrane was performed with UV (2X autocrosslinking, UV Stratalinker 1800). The oligonucleotides primers (Supplementary Table I) were labelled with gamma-^32^P ATP (Hartmann analytics) using the T4 Polynucleotide Kinase (T4-PNK, Fermentas) and purified over G-25 columns (GE Healthcare) as previously described [33]. The denatured primers were incubated with the membranes in hybridization buffer (Rapid-hyb buffer, GE Healthcare) overnight at 50°C. The membranes were washed with 1X SSC + 0.1% SDS for 20 min at 50°C and subsequently with 0.5X SSC + 0.1% SDS. The radioactive signal was visualized after 2 or 3 days of exposure using a phosphorimager (Typhoon Fla 9000, Fujifilm). 16S rRNA was used as a loading control. The approximate sizes of RNA transcripts were estimated using the RiboRuler High Range Ladder (Thermo Scientific). Each experiment was performed at least in independent triplicates.

### Calculation of RNA half-life

The transcript abundance quantification was performed by densitometry using Fiji [50]. The half-lives were calculated by fitting an exponential decay curve to time points measured from three independent blots. The comparison of the half-lives between WT and ∆*rny* or between ∆*rny::rny* and ∆*rny* was done using a t-test.

### Primer extension

Primer extension was performed as described in [43]. The sizes of cDNA fragments were estimated using the 30–330-bp AFLP® DNA ladder (Invitrogen). Alternatively, a sequencing ladder was generated with a dideoxy chain termination reaction, using the Sequenase^TM^ Version 2.0 DNA Sequencing Kit (Affymetrix USB), following the manufacturer’s instructions. The DNA template used during the termination reaction was generated by PCR from WT genomic DNA using OLEC3903/OLEC3972.

### Luminescence assay for *speB* promoter activity

The plasmid-based reporter system (pLZ12Km2-P23R:TA:*ffluc*, Addgene plasmid gift from Thomas Proft) described in [51] was used to construct plasmid pEC2248, in which the expression of *ffluc* (firefly luciferase gene) is under the control of the *speB* promoter region (from –940 nt to –697 nt relative to the *speB* start codon). This region contains both P and P1 promoters (P*_speB_*) and the putative binding sites of *speB* transcriptional regulator RopB [23,26]. Briefly, pLZ12Km2-P23R:TA:*ffluc* was digested with SacI and SacII (Thermo Scientific) to remove the lactococcal constitutive promoter P23 [51,52]. P*_speB_* was amplified from WT genomic DNA using primers OLEC8386/OLEC8387 and cloned in pLZ12Km2-P23R:TA:*ffluc*. WT, ∆*rny*, and ∆*rny::rny* cells containing the constructs for studying the activity of *speB* promoters were grown until ML and ES growth phases. Beetle luciferin potassium salt (Promega) was added to 200 μl of cultures at a final concentration of 50 ng/μl in a white opaque 96-well microtiter plate (Greiner Bio-One^TM^). Luminescence was measured using a microplate reader (BioTek^TM^ Cytation 3) with an integration time of 1 sec, a gain of 120, and a read height of 1 mm. The signal was normalized with the OD_620_ _nm_ and with the luciferase signal obtained from the constitutive promoter P23. The experiments were carried out in independent biological triplicates, each with technical triplicates.

### Exoprotein precipitation

*S. pyogenes* cultures were grown until ML and ES growth phases, centrifuged and subsequently filtered using 0.45 μm syringe filter (VWR) to obtain cell-free supernatants. The proteins were precipitated with 10% of ice-cold trichloracetic acid and resuspended in 70 μl of 1 M Tris-HCl, pH 8.0. Equal volumes of exoprotein preparation were separated by SDS-PAGE (sodium dodecyl sulfate – polyacrylamide gel electrophoresis) using 15% polyacrylamide gel. The gels were stained with Coomassie Brilliant Blue (Sigma-Aldrich) and imaged using CanoScan LiDE 700F. The approximate masses in kilodaltons (kDa) of the proteins were estimated using PageRuler^TM^ Plus Prestained Protein ladder (Thermo Fisher).

### RNA secondary structure prediction

The RNA structure of *speB* 5′ UTR (Fig. S3A) was estimated by calculating the minimum free energy (RNAfold, MFE in Kcal/mol) [53] of 25 nt sequences at each position from 100 nt upstream to 100 nt downstream of the RNase Y cleavage site (at –137 nt from *speB* ATG). The structure prediction (Fig. S3B) was visualized by using VARNA [54].

### RNA sequencing

Total RNA was extracted from *S. pyogenes* WT grown until ES phase for RNA sequencing or until ML phase for sRNA sequencing, and DNA was removed using Turbo DNase (Ambion). After sample quality control using the bioanalyzer (RNA 6000 Nano Kit) and Qubit (RNA BR Assay, Invitrogen), the rRNAs were removed with Ribo-Zero rRNA Removal Kit Bacteria (Illumina). To include both primary and processed transcripts in the libraries, the 5′ triphosphate RNAs were converted in 5′ monophosphate RNAs using TAP (Tobacco Acid Pyrophosphatase, Epicentre). After treatment with T4 Polynucleotide kinase (Thermo Scientific), small and large RNAs were purified into two separated fractions (short and long RNAs) using RNA Clean & Concentrator kit (Zymo Research). RNAs fragments of 200 nt were further produced from the long RNA fraction by chemical fragmentation with NEBNext Magnesium RNA Fragmentation Module (NEB E61505). RNA purification steps were done using Phenol:Chloroform:Isoamylalcohol (Roth) followed by Chloroform:Isoamylalcohol (Sigma) or RNA Clean & Concentrator kit (Zymo Research). The cDNA libraries were prepared using NEXTflex® Small RNA Sequencing Kit v3 (Bioo Scientific) for both short and fragmented RNA fractions, following the manufacturer’s instructions with some modifications. After step G, consisting of 22 cycles of PCR, the final purification of the cDNA library was done with Agencourt AMPure XP beads (Beckman Coulter), following step I of the NEXTflex Rapid Directional qRNA-Seq Kit protocol (Bioo Scientific). cDNA library quality control was checked using Qubit (dsDNA HS Assay, Invitrogen) and bioanalyzer (High sensitivity DNA kit, Agilent). The sequencing was performed using the HiSeq3000 instrument at the Max Planck-Genome-Centre Cologne (MP-GC), with a paired end strategy and 150 nt of read length.

### Read processing

FastQC (v0.11.5) was used to assess the quality of the data. Reads with quality score <10 were filtered and eliminated if smaller than 22 nt and adapter sequences were removed using Cutadapt (v1.11) [55]. The mapping of the reads against the *S. pyogenes* reference genome (NC_002737.2) was performed with STAR (v2.5.2b) in both ‘random best’ and ‘end to end’ modes [56]. The resulting BAM files were sorted and indexed using Samtools (v1.3.1). To detect and filter possible PCR artefacts, four random bases UMIs (Unique Molecular Identifiers) were included at the RNA 3′ and 5′ ends, during the library preparation and treated with the UMI Tools (v0.4.1). The coverage files (total, 5ʹ and 3ʹ ends) were produced using a custom script with the HTSeq library (v0.9.1) and visualized using the Integrative Genomics Viewer (IGV) [57].

## Data Availability

The RNA sequencing data have been deposited at NCBI under the accession number SRP149897.
