# Supplementary material for: RNase Y-mediated regulation of the streptococcal pyrogenic exotoxin B
Source: RNA Biol. 2018 Oct 18;15(10):1336–47. doi: 10.1080/15476286.2018.1532253 (PMC6284565; doi:10.1080/15476286.2018.1532253)
Supplement: Supplemental Material [file krnb-15-10-1532253-s001.pdf]

Supplementary materials for:

RNase Y-mediated regulation of the streptococcal pyrogenic exotoxin B

Laura Broglia[1,2,3,4], Solange Materne[1,2], Anne-Laure Lécrivain[1,2,5], Karin Hahnke[1,2], Anaïs Le Rhun[1,2,4,5*] and Emmanuelle Charpentier[1,2,3,4,5*]

[1]Max Planck Unit for the Science of Pathogens, D-10117 Berlin, Germany

[2]Max Planck Institute for Infection Biology, Department of Regulation in Infection Biology, D-10117 Berlin, Germany

[3]Institute for Biology, Humboldt University, D-10115 Berlin, Germany

[4]Helmholtz Centre for Infection Research, D-38124 Braunschweig, Germany

[5]The Laboratory for Molecular Infection Medicine Sweden (MIMS), Umeå Centre for Microbial Research (UCMR), Department of Molecular Biology, Umeå University, S-90187 Umeå, Sweden

[*]Co-corresponding authors

Correspondence:

Anaïs Le Rhun, Email: lerhun@mpiib-berlin.mpg.de

Emmanuelle Charpentier, Email: research-charpentier@mpiib-berlin.mpg.de

Supplementary materials include:

Supplementary Figures

Supplementary Table I

Supplementary Table II

Supplementary Figures

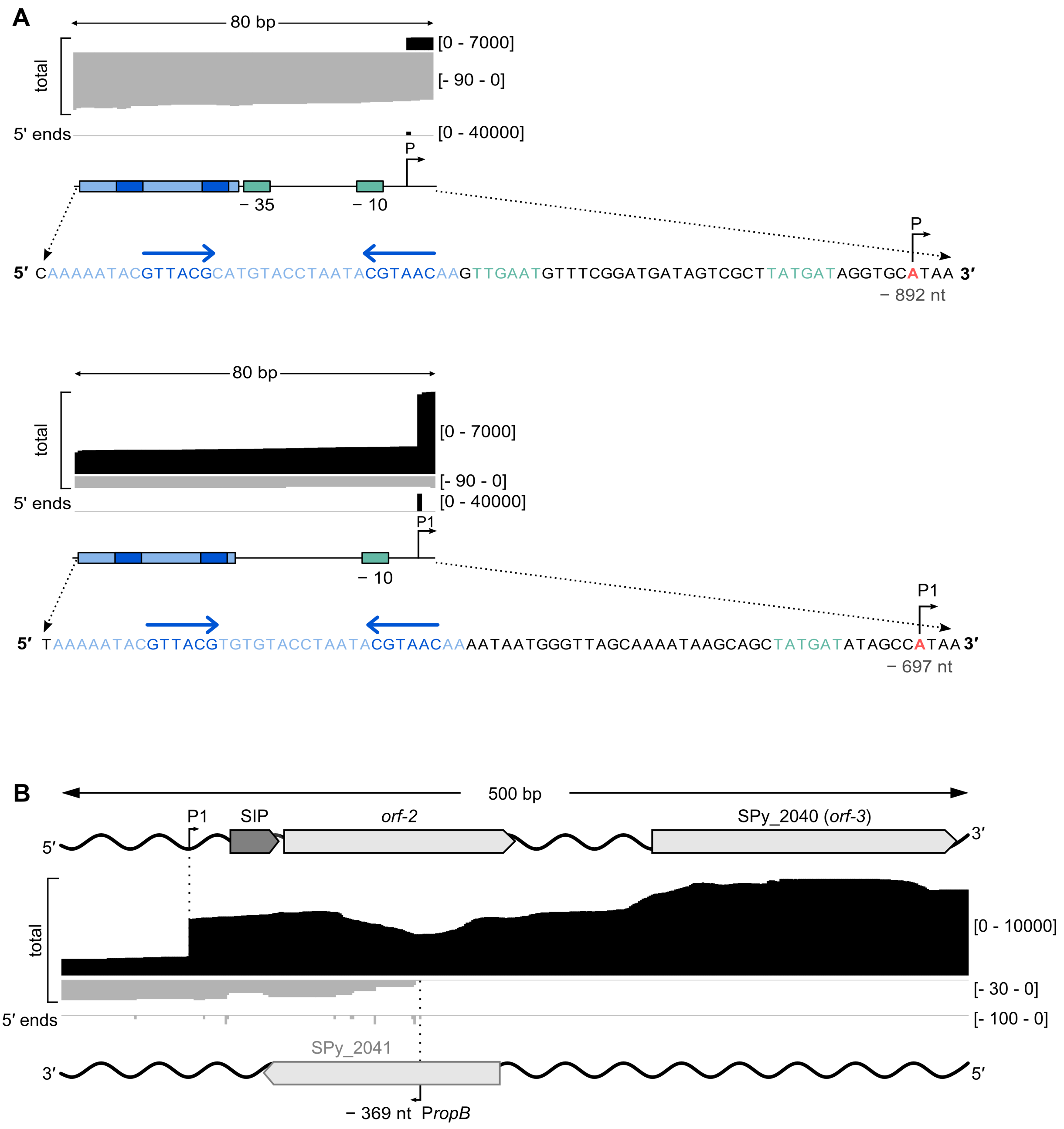

Figure S1. *ropB-speB* intergenic region

A-B. Total and 5′ end coverages (black for positive strand, grey for negative strand) are indicated between brackets.

A. Zoom on *speB* promoters, P (top panel) and P1 (bottom panel). The predicted − 35 and – 10 motifs were mapped for P and P1. The putative RopB binding sites, consisting

of inverted repeats (dark blue boxes and arrows) located within direct repeats (light blue boxes), are annotated upstream of P and P1 [1,2]. **B.** Characterization of the *ropB*-*speB* intergenic region by RNA sequencing analysis. The *ropB* (P_{ropB}) and *speB* (P1) TSSs are shown with black bent arrows. P_{ropB} is located – 369 nt relative to the *ropB* start codon (not indicated here). In the experimental conditions used in this study, SPy_2041 is not transcribed.

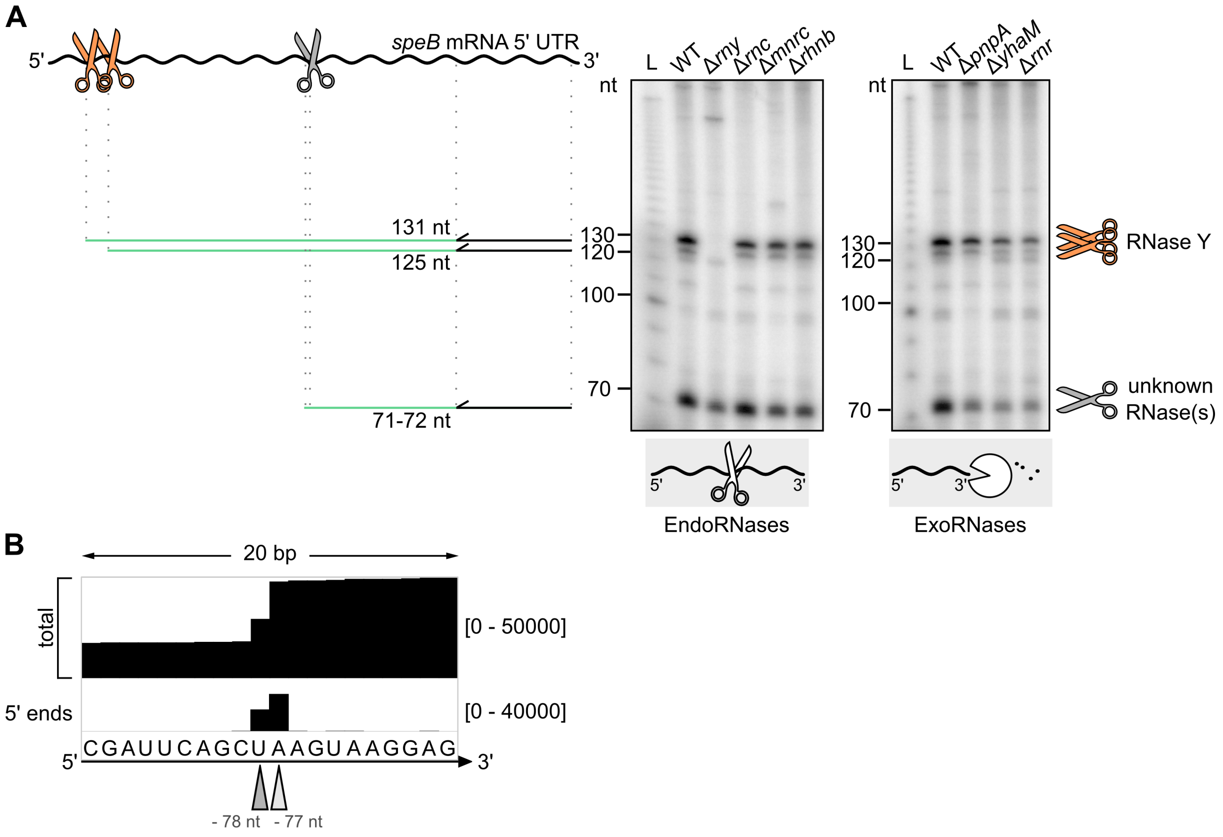

Figure S2. Unknown RNase(s) process the *speB* mRNA 5′ UTR

A. Schematic drawing of *speB* mRNA 5′ UTR. Processing by RNase Y (orange scissors) and unknown RNase(s) (grey scissors) are indicated (left panel). The primer used (black arrow) for primer extension (right panel) and the expected cDNA sizes (green lines) are depicted. The processed 5′ ends of *speB* mRNA 5′ UTR were identified using primer extension analyses (right panel) in WT, *rny* (RNase Y) deletion mutant (Δ*rny*), *rnc* (RNase III) deletion mutant (Δ*rnc*), *mrnc* (Mini-III) deletion mutant (Δ*mrnc*), *rhnb* (RNase HII) deletion mutant (Δ*rhnb*), *pnpA* (PNPase) deletion mutant (Δ*pnpA*), *yhaM* (YhaM) deletion mutant (Δ*yhaM*) and *rnr* (RNase R) deletion mutant (Δ*rnr*) at early-stationary growth phase. **B.** Zoom on the processing sites (grey triangles) of *speB* mRNA 5′ UTR at positions − 77 nt and − 78 nt (relative to the *speB* start codon) retrieved by RNA sequencing analysis. The total and the 5′ end coverages are indicated between brackets.

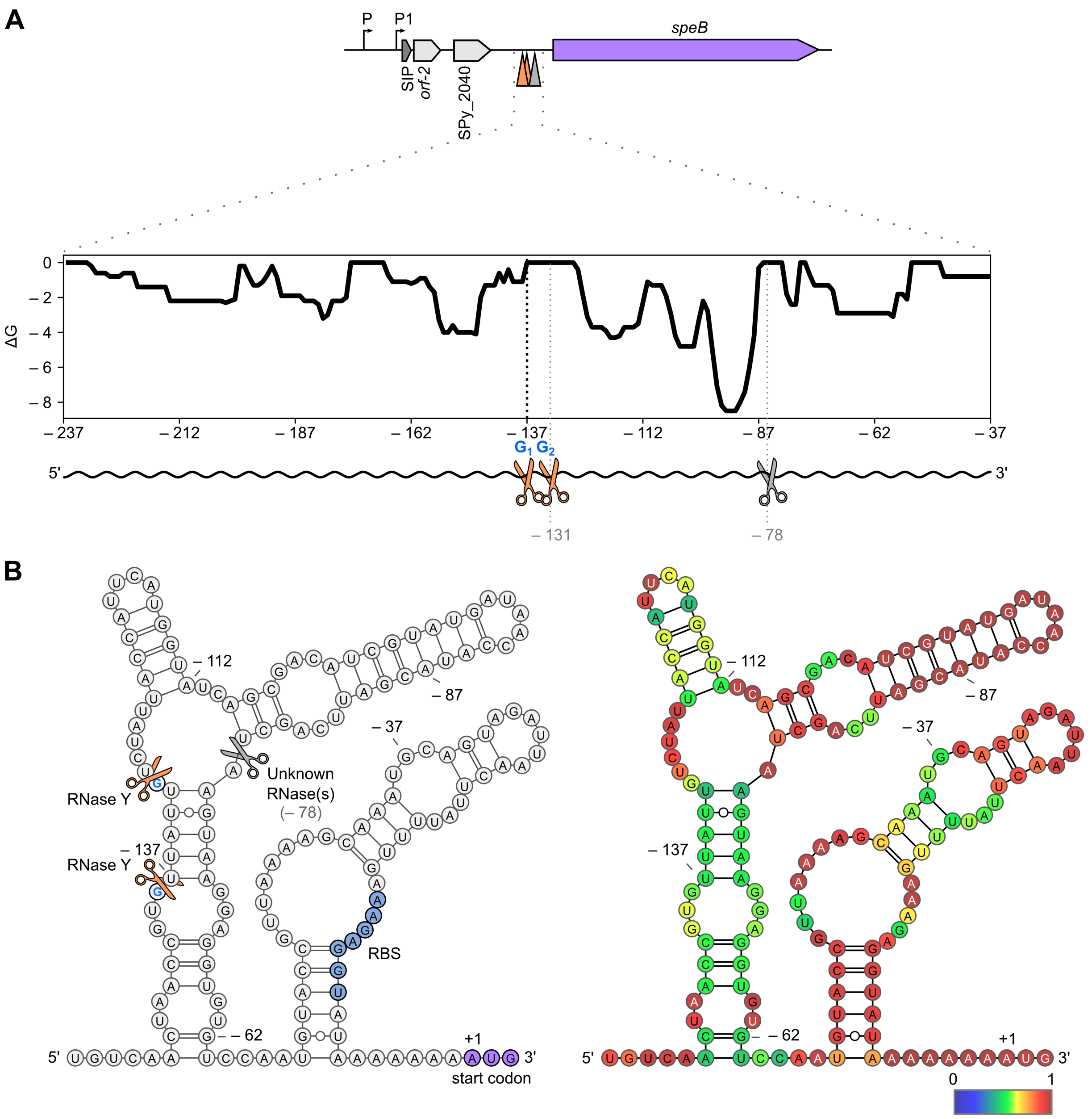

Figure S3. Secondary structure prediction of the *speB* mRNA 5′ UTR

A. Schematic drawing of *speB* mRNA 5′ UTR (containing SpeB Inducing Peptide (SIP), *orf2*, SPy_2040) and *speB* coding DNA sequence (CDS). The positions corresponding to the cleavage sites of RNase Y and of unidentified RNase(s) are represented with orange and grey triangles, respectively. The two Gs located upstream of the RNase Y processing sites at positions – 137 nt (G_1) and – 131 nt (G_2) are indicated. The minimal folding energy (MFE, ΔG in Kcal/mol) was calculated both 100 nt upstream and downstream of the RNase Y cleavage site (– 137 nt). The numbers indicate the

distance in nt to *speB* start codon. **B.** RNA folding of a portion of *speB* 5′ UTR (from position – 153 nt to the *speB* start codon). The free energy of the thermodynamic ensemble is – 31.48 kcal/mol. The cleavages by RNase Y and unidentified RNase(s) are indicated by orange and grey scissors, respectively (right panel). The *speB* ribosome binding site (RBS) and start codon are represented in purple (left panel). The same structure was colored by base-pairing probabilities (right panel). The color of the unpaired regions indicates the probability of being unpaired.

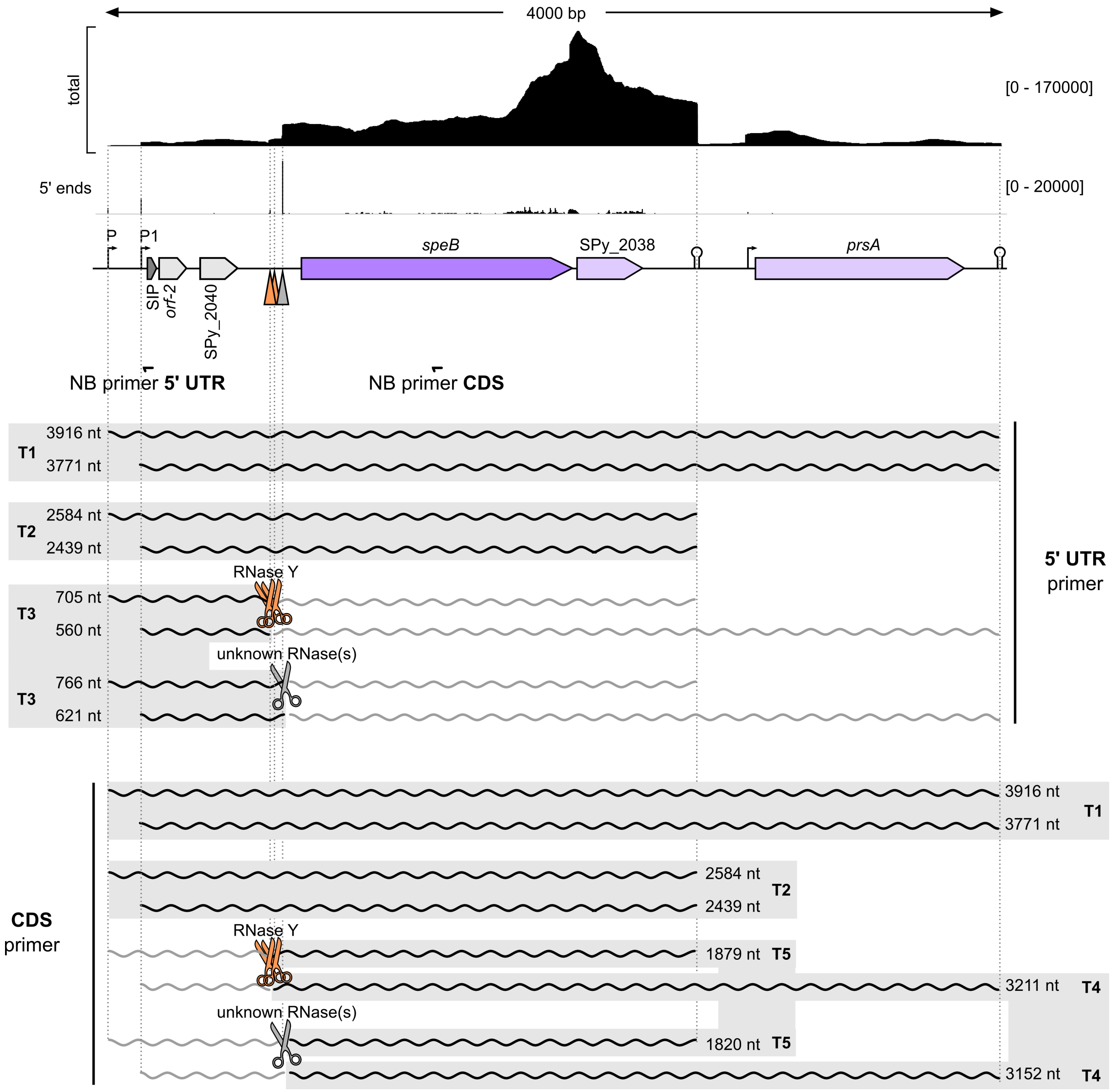

Figure S4. Isoforms of *speB* mRNA

Expression profile of *speB* locus and surrounding genes resulting from RNA sequencing analysis. The 5′ ends retrieved are depicted with black bars. The genes (arrows) with the putative promoters (P and P1) and terminators are indicated. Putative ORFs (SPy_2040 and *orf-2*) and the sequence encoding the SpeB Inducing Peptide (SIP) are annotated in the *speB* 5′ UTR. *speB* is co-transcribed with the SPy_2038 and *prsA* genes [3]. The cleavages by RNase Y and by unknown RNase(s) are depicted with orange and grey triangles, respectively. The primers used in the Northern blot

analyses (Figure 4A and 4B) are indicated below the locus. The expected transcript isoforms detectable with the primers targeting the 5′ UTR (T1, T2 and T3) (Figure 4A) and the CDS (T1, T2, T4, T5) (Figure 4B) are shown as black curved lines and the sizes in nt are indicated.

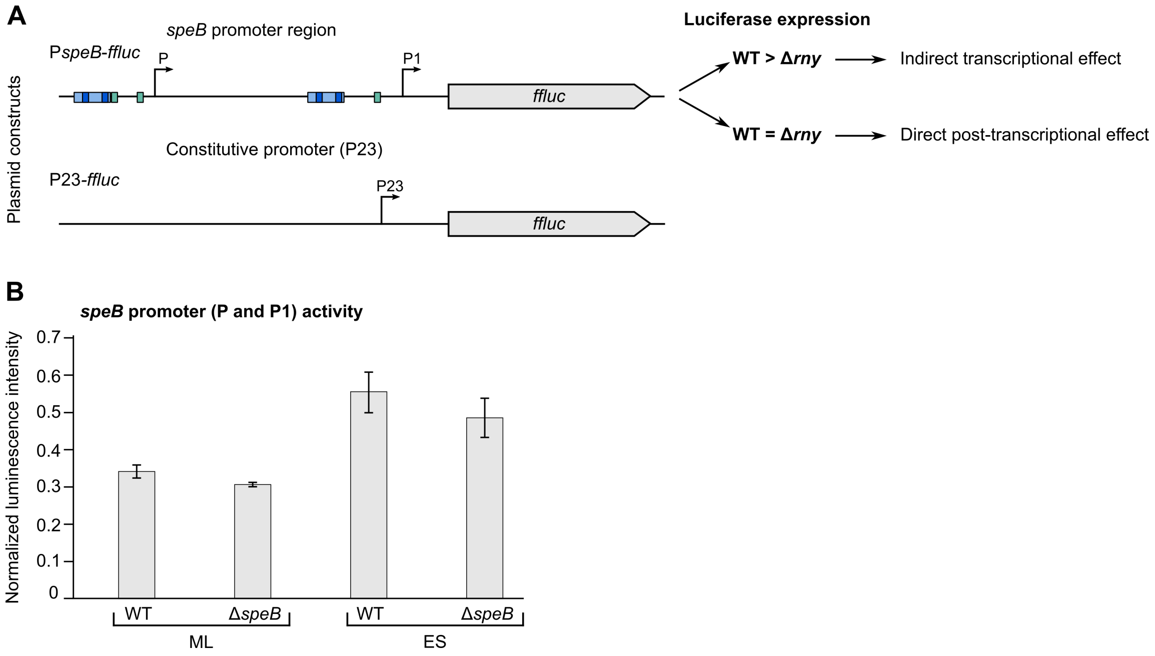

Figure S5. Study of *speB* promoter activity

A. Schematic representation of luciferase (*ffluc*) fusion plasmids used in Figure 4A and S4B. The *speB* promoters were cloned upstream of the *ffluc* gene (P*speB*-*ffluc*). The – 10 and – 35 motifs of P and P1 are depicted with green boxes. The putative RopB binding sites are indicated in blue. A control vector with *ffluc* expression under the control of a constitutive promoters (P23) was included in the analysis (P23-*ffluc*). **B.** The *speB* promoter activity was examined by luminescence assay performed in the WT and *speB* deletion mutant (Δ*speB*) containing the luciferase fusion plasmids (P23-*ffluc* and P*speB*-*ffluc*) at mid-logarithmic (ML) and early-stationary (ES) growth phases. Values indicate luminescence intensity of the samples relative to the control plasmid (P23-*ffluc*), normalized to the $OD_{620\ nm}$. Mean and standard deviations (error bars) were calculated from three independent experiments, each with technical triplicates.

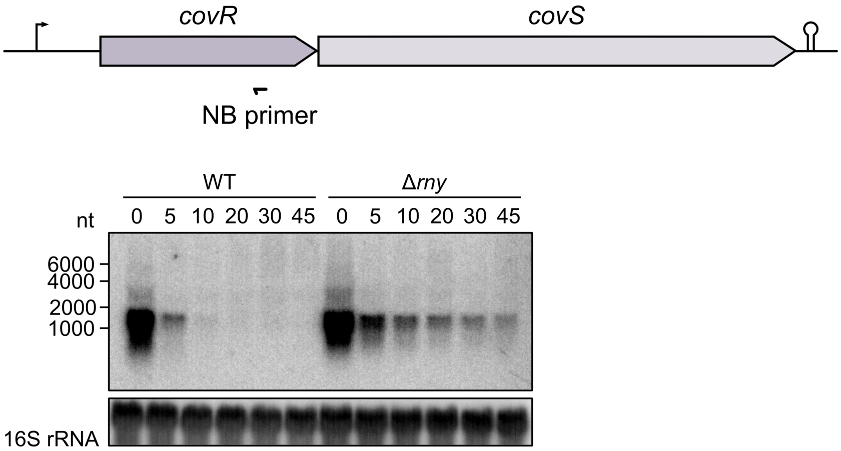

Figure S6. *covR* mRNA stability is affected by RNase Y

Study of the *covR* transcript stability by rifampicin assay at mid-logarithmic phase of growth in WT and *rny* (RNase Y) deletion mutant (Δ*rny*) (lower panel). The minutes after stopping transcription upon the addition of antibiotic are indicated. 16S rRNA was used as a loading control. The primer used is indicated by a black arrow.

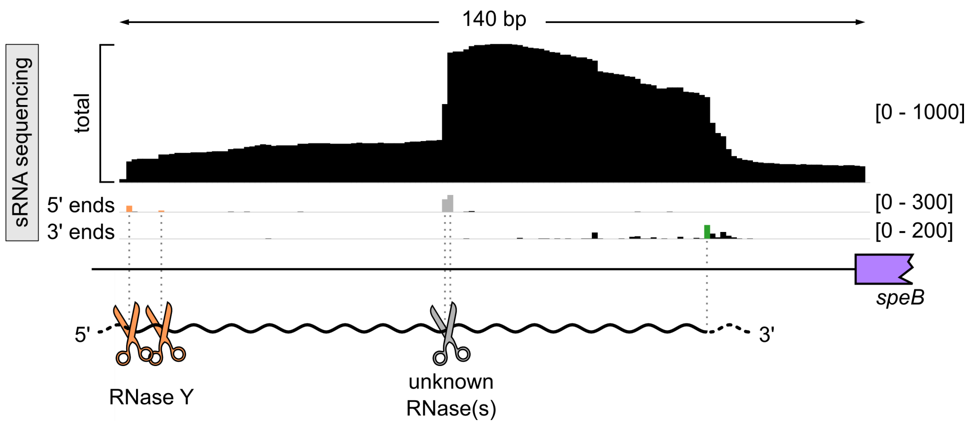

Figure S7. An sRNA arises from *speB* mRNA 5′ UTR processing

Expression profile of a small RNA (sRNA) previously identified in *speB* 5′ UTR by sRNA sequencing (Spy_sRNA1699993) [4]. Total, 5′ end and 3′ end coverages are indicated between brackets. Orange and grey bars pinpoint the positions of RNase Y and unidentified RNase(s) cleavage sites annotated in this study, respectively. The green bar denotes the putative sRNA 3′ end.

Supplementary Table I. Strains, plasmids and oligos used in this study.

Strain	Relevant characteristics	Source
Streptococcus pyogenes		
**WT**		
EC2224	SF370 (M1 serotype)	ATCC 700294
**Δ*rny***		
EC2246	EC2224Δ*rny*::lox72	[5]
**Δ*rnc***		
EC2249	EC2224Δ*rnc*::lox72	[5]
**Δ*rnr***		
EC2254	EC2224Δ*rnr*::lox72	This study
**Δ*pnpA***		
EC2297	EC2224Δ*pnpA*::lox72	This study
**Δ*rny*::*rny***		
EC2298	EC2246Δlox72::*rny*-TT3-lox72	This study
**Δ*yhaM***		
EC2347	EC2224ΔSPy_0267::lox71- P*ermAM/B*-*ermAM/B*-lox66	This study
**Δ*rnhB***		
EC2251	EC2224Δ*rnhB*::lox72	This study
**Δ*mrnc***		
EC2271	EC2224Δ*mrnc*::lox72	This study
**Δ*speB***		
EC2356	EC2224Δ*speB*::lox72	This study
Saccharomyces cerevisiae		
S228C	BY4741 (Host for cloning)	Euroscarf, Frankfurt
Escherichia coli		
RDN204	Top10 (Host for cloning)	Invitrogen

Plasmid	Relevant characteristics	Source
**Plasmids used for gene deletion in *S. pyogenes***		
pEC454	pUC19Ωlox71-*ermAM/B*-lox66	Laboratory collection
pEC455	pEC85ΩP*gyrA*-*cre*	Laboratory collection
pEC707	pUC19, pMB1, *ampR*	New England Biolabs

pEC748	pUC19Ω*rnhB*::lox71-*PermAM/B-ermAM/B*-lox66	This study
pEC749	pUC19Ω*mrnc*::lox71-*PermAM/B-ermAM/B*-lox66	This study
pEC801	pSEVA141, pRO1600/ColE1, *ampR*	de Lorenzo´s lab
pEC2145	pEC801Ω*speB*::lox71-P*ermAM/B-ermAM/B*-lox66	This study
pEC545	pJET1.2Ω*rnr*koup-lox71-P*ermAM/B-ermAM/B*-lox66-*rnr*kodw	This study
pEC750	pEC707Ω*pnpA*koup-lox71-P*ermAM/B-ermAM/B*-lox66-*pnpA*kodw	This study
pEC822	pEC801ΩSPy_0267koup-lox71-P*ermAM/B-ermAM/B*-lox66-SPy_0267kodw	This study
**Chromosomal complementation of *rny* in *S. pyogenes***		
pEC802	pRS426Ω*rnyup-rny*-TT3-lox71-P*ermAM/B-ermAM/B*-lox66-*rny*dw	This study
speB* ectopic expression in *S. pyogenes		
pEC85	*repDEG*-pAMß1, *aphIII*-Pjh1, ColeE1	Laboratory collection
pEC2146	pEC85ΩP*gyrAspeB*	This study
pEC2249	pEC85ΩP*gyrA-speB*(G-137A)	This study
pEC2250	pEC85ΩP*gyrA-speB*(G-131A)	This study
pEC2263	pEC85ΩP*gyrA-speB*(G-137A_G-131A)	This study
pEC2264	pEC85ΩP*gyrA-speB*(∆-147-121)	This study
pEC2265	pEC85ΩP*gyrA-speB*(∆-157-111)	This study
**Luminescence assay in *S. pyogenes***		
pEC2173	pLZ12Km2-P23R:TA:*ffluc*	Addgene plasmid # 88900
pEC2248	pEC2173ΩP*speB*	This study

Oligo	Sequence 5′-3′ [a]	F/R [b]	Usage [c]	Target[d]
**∆*rny*Ω*rny***				
OLEC3584	GTAACGCCAGGGTTTTCCCAGTCACGAC*GCTCTTCAAACGAAAAAGAGG*	F	Cloning	Up fragment (pEC802)
OLEC3579	CGAGAAAAAAGGCCCACTTTTGTGGGCCTTTTTTACGCAAGAAGCCAC*TACTTGGCATAATCAACCGCTCTCATCTC*	R	Cloning	
OLEC3480	AAGTGGGCCTTTTTTCTCGGAT*TACCGTTCGTATAGCATACATTATACGAAGTTATCCG*	F	Cloning	lox71-*ermAM/B*-lox66 (pEC454)
OLEC3572	*TACCGTTCGTATAATGTATGCTATACGAAGTTATTTATTTCCTCCCGTTAAATAATAGATAAC*	R	Cloning	
OLEC2000	ATAGCATACATTATACGAACGGTA*AAAAAGAGGAATTATCCTCTTTTTCTTTATGA*	F	Cloning	Down fragment (pEC802)
OLEC3585	GCGGATAACAATTTCACACAGGAAACAG*CGTAAAATCACAAGTGAATACTTGG*	R	Cloning	
OLEC2785	*TCGCAATCGTTGAAAATCAT*	F	PCR, SEQ	Upstream *rny*
OLEC2503	*GACAGCTTCACGTTTAGCTGAAG*	R	PCR, SEQ	Downstream *rny*
**∆*rnhB***				
OLEC3340	GGTGGTGGATCC*CGAAGTGAAGCTAATCATGC*	F	Cloning	Up fragment (pEC748)
OLEC2517	TATAATGTATGCTATACGAACGGTA*ATACTAGTCGGCATCCATATCTCC*	R	LM-PCR	
OLEC2518	ATAGCATACATTATACGAACGGTA*TAAAAAGTTCTGTTTTTAGCAGAATTTTTTTCTTTT*	F	LM-PCR	Dw fragment (pEC748)
OLEC3341	GGTGGTGGATCC*TGGGACAGCAAAAATGTCTCG*	R	Cloning	

Oligo	Sequence 5′-3′ [a]	F/R [b]	Usage [c]	Target[d]
OLEC2520	*TTGCAAGCAAAAACTGTAAAGACTTAAAAG*	F	SEQ	Upstream *rnhB*
OLEC2521	*CATAATATCCCATTTTTAAGAAACTGTCAATA*	R	SEQ	Downstream *rnhB*
**Δ*mrnc***				
OLEC2034	GATGATGGATCC*CCCTGTCAGAACTTGAAGTTGGAG*	F	Cloning	Up fragment (pEC749)
OLEC3353	ATAGCATACATTATACGAACGGTA*AATTCACATCAACTGGATTA GTCAC*	R	LM-PCR	
OLEC3352	TATAATGTATGCTATACGAACGGTA*CATAGGTCTGAAGTAAAGG TAGAGAG*	F	LM-PCR	Dw fragment (pEC749)
OLEC2033	GGTGGTGAGCTC*CAATAGTATCTTTATCTTCCATGAG*	R	Cloning	
OLEC2005	*CCTCGTGTTATGGATTATATAGCA*	F	SEQ	Upstream *mrnc*
OLEC2006	*AGGCGTCCATGAAATAGCGACCTT*	R	SEQ	Downstream *mrnc*
**Δ*speB***				
OLEC7565	AAAGGATCC*ATGTCAAAAATACGTTACGCATG*	F	Cloning	Up fragment (pEC2145)
OLEC7566	TATAATGTATGCTATACGAACGGTA*TTTTTTTATACCTCTTTCAA AATAAGTTAATCTAC*	R	LM-PCR	
OLEC7902	ATAGCATACATTATACGAACGGTA*GACGGACGTAACTTCTACCA TGTT*	F	LM-PCR	Dw fragment (pEC2145)
OLEC7569	*AAAGGATCCTGTTGTGTGATGATTGACAAGCTG*	R	Cloning	
OLEC7563	*TGAATGCCTAATGAATTCAACGG*	F	PCR, SEQ	Upstream *speB*
OLEC7570	*GTGTTTTTGGTCTCATTGTAGAAGT*	R	PCR, SEQ	Downstream *speB*
**Δ*rnr***				
OLEC2897	AAAGGATCC*GGAGATCGATTTGGCAATCA*	F	Cloning	Up fragment (pEC545)
OLEC2535	TATAATGTATGCTATACGAACGGTA*ACCTAATTTCT ATTTCTGTTTTGTTTGTTGG*	R	LM-PCR	
OLEC2536	ATAGCATACATTATACGAACGGTA*AAAAAGAAGAG TCGTAAAAGGAGTTAACT*	F	LM-PCR	Dw fragment (pEC545)
OLEC2898	AAAGGTACCATCTTTGGGGTCTCGCTTTT	R	Cloning	
OLEC2538	*CTCACAACTTAATGTTTACTTCAGGC*	F	PCR, SEQ	Upstream *rnr*
OLEC2539	*TATTGGCATAGAGATAACCATCTACATA*	R	PCR, SEQ	Downstream *rnr*
**Δ*pnpA***				
OLEC3350	GCTAGGATCC*CAGTTCTTATATTGGCTTTGCC*	F	Cloning	Up fragment (pEC750)
OLEC2541	TATAATGTATGCTATACGAACGGTA*ATATTCTCCTTT TAATTTTCAGAGGGG*	R	LM-PCR	
OLEC2542	ATAGCATACATTATACGAACGGTAG*AAAAAAGAAGA AAAACATGACTAAATCAAATGAA*	F	LM-PCR	Dw fragment (pEC750)
OLEC3351	GCTAGGATCC*CTTTGATGCCTGGATAAGTTAGG*	R	Cloning	
OLEC2544	*CTAAACGTTAAAGTCTTTTCAGACGGT*	F	PCR, SEQ	Upstream *pnpA*
OLEC2545	*ATGAAGACTCCAGGAGCGATTTG*	R	PCR, SEQ	Downstream *pnpA*
**Δ*yhaM*** (**ΔSPy_0267**)				
OLEC3361	GAAGCTGCAG*CCTCTTTCGATTCTGTATCC*	F	Cloning	Up fragment (pEC822)
OLEC2529	TATAATGTATGCTATACGAACGGTA*TTAATTTTCATT ATTTTCCTCTTCTAATAAGGG*	R	LM-PCR	
OLEC2530	ATAGCATACATTATACGAACGGTA*TGATCAGTGTTT CTCGAGTAATAGTTC*	F	LM-PCR	Dw fragment (pEC822)
OLEC3362	GAAGGTCGAC*GCATTGGCAATAATACGACC*	R	Cloning	
OLEC2532	*GACCGGTCTGACAAACGCTTA*	F	PCR, SEQ	Upstream SPy_0267
OLEC2533	*GTCATTTGCTCACGCTCTGATTG*	R	PCR, SEQ	Downstream SPy_0267
**pEC2146**				
OLEC7968	CCTTTCTAGA*CTATCATTTTCAATGAAAGAAGTCACTAATAAAAT GTGA*	F	Cloning	P*gyrA* (pEC455)
OLEC7969	CATAGTAGGCGCCTCCTTTTAACCTTAT*TACATTGTACCATAATT TAGGTAAAATTGCGATGAT*	R	LM-PCR	
OLEC7970	ATCATCGCAATTTTACCTAAATTATGGTACAATGTA*ATAAGGTTA AAAGGAGGCGCCTACTATG*	F	LM-PCR	*speB*
OLEC7971	CCCAGAATTC*CTAAGGTTTGATGCCTACAACAGCAC*	R	Cloning	

Oligo	Sequence 5′-3′ [a]	F/R [b]	Usage [c]	Target[d]
**pEC2249**				
OLEC8388	*GTCAACTAACCGTATTATTGTCTATTACCAT*	F	TS-PCR	*speB* 5′ UTR (pEC2146)
OLEC8389	*GTCAACTAACCGTATTATTGTCTATTACCAT*	R	TS-PCR	
**pEC2250**				
OLEC8390	*GTCAACTAACCGTGTTATTATCTATTACCAT*	F	TS-PCR	*speB* 5′ UTR (pEC2146)
OLEC8391	*ATGGTAATAGATAATAACACGGTTAGTTGAC*	R	TS-PCR	
**pEC2263**				
OLEC8392	*GTCAACTAACCGTATTATTATCTATTACCAT*	F	TS-PCR	*speB* 5′ UTR (pEC2146)
OLEC8393	*ATGGTAATAGATAATAATACGGTTAGTTGAC*	R	TS-PCR	
**pEC2264**				
OLEC8394	*GTTGGGTTGTCAGTGTCATCATGGTATCAGCGACAT*	F	TS-PCR	*speB* 5′ UTR (pEC2146)
OLEC8395	*ATGTCGCTGATACCATGATGACACTGACAACCCAAC*	R	TS-PCR	
**pEC2265**				
OLEC8396	*GAATAATTGGGTTGGGTTAGCGACATCGTATGATAA*	F	TS-PCR	*speB* 5′ UTR (pEC2146)
OLEC8397	*TTATCATACGATGTCGCTAACCCAACCCAATTATTC*	R	TS-PCR	
**pEC2248**				
OLEC8386	CGAGCTC*ATGTCAAGCCTTCCTAGTTGATGTCA*	F	Cloning	*speB* 5′ UTR
OLEC8387	TACCCGCGG*TGGCTATATCATAGCTGCTTATTTTGCT*	R	Cloning	
**Sequencing**				
OliRN228	*GGAACGAAAACTCACGTTAA*	F	SEQ	pEC85
OLEC787	*TGTGGTTACGTGGTTTTTAAC*	R	SEQ	
OLEC3224	*TGTAAAACGACGGCCAGT*	F	SEQ	pEC707 pEC2173
OLEC3225	*CAGGAAACAGCTATGACC*	R	SEQ	
OLEC3600	*CCAGGGTTTTCCCAGTCACGAC*	F	SEQ	pEC801
OLEC3590	*AGCGGATAACAATTTCACACAGGA*	R	SEQ	
OLEC1938	*TCAATCGAGAATATCGTCAACTGTTTACTAAA*	F	SEQ	*ermAM/B*
OLEC1937	*TTGCTGTTTCGATTTTTATGATATGGTGC*	R	SEQ	
OLEC5336	*GGGGGATGTGCTGCAAGGCG*	F	SEQ	pEC802
OLEC5337	*TCCGGCTCCTATGTTGTGTGG*	R	SEQ	
**Primer extension analyses**				
OLEC2406	*ACTACCATTTTGCAAAAGGAAC*	R	PE	*speB* 5′ UTR
OLEC3903	*TAACGGTACATTGGACACACCTCC*	R	PE	
OLEC3904	*TATACCTCTTTCAAAATAAGTTAATCTACTGC*	R	PE	
OLEC3970	*TGGGTTAGCAAGAACAAATCC*	R	PE	*speB* CDS
**Northern blot analyses**				
OLEC5802	*AACCACATAGTAGGCGCCTC*	R	NB	*speB* 5′ UTR
OLEC7431	*GCAACACATCCTGTAGCTGC*	R	NB	*speB* CDS
OLEC1542	*CATGACACGATTCATATTAGTC*	R	NB	*covR* CDS
OliRN243	*CGTTGTACCAACCATTGTAGC*	R	NB	16S rRNA

[a] *italic*: sequence annealing to the template; underlined: restriction site.

[b] F: forward primer; R: reverse primer.

[c] LM-PCR: ligation-mediated PCR; TS-PCR: two-stage PCR; SEQ: sequencing; PE: primer extension; NB: Northern blot;

[d] 5′ UTR: 5′ untranslated region; CDS: coding DNA sequence

Supplementary Table II. *speB* regulators potentially affected by RNase Y.

***speB* regulators**	**Function**	**References**
**Direct transcriptional regulators**		
ropB	Activator	[1,6–8]
covRS	Repressor	[9–11]
ccpA	Activator	[11–13]

***speB* regulators**	**Function**	**References**
**Indirect transcriptional regulators via RopB**		
**LacD.1**	Repressor	[14]
vfr	Repressor	[15,16]
**SIP**	Activator	[2,17]

Except for *vfr* abundance [18] and *ropB* stability [19], which were shown to be affected by RNase Y, the effect of RNase Y on the other regulators is to be confirmed [20]. SpeB Inducing Peptide (SIP) is encoded by the *speB* transcript, and therefore its expression is downregulated in the *rny* deletion strain.

References

[1] Neely MN, Lyon WR, Runft DL, et al. Role of RopB in growth phase expression of the SpeB cysteine protease of *Streptococcus pyogenes*. J Bacteriol. 2003;185:5166–5174.

[2] Do H, Makthal N, VanderWal AR, et al. Leaderless secreted peptide signaling molecule alters global gene expression and increases virulence of a human bacterial pathogen. Proc Natl Acad Sci USA. 2017;114:E8498–E8507.

[3] Ma Y, Bryant AE, Salmi DB, et al. Identification and characterization of bicistronic *speB* and *prsA* gene expression in the Group A Streptococcus. J Bacteriol. 2006;188:7626–7634.

[4] Le Rhun A, Beer YY, Reimegård J, et al. RNA sequencing uncovers antisense RNAs and novel small RNAs in *Streptococcus pyogenes*. RNA Biol. 2016;13:177–195.

[5] Le Rhun A, Lécrivain A-L, Reimegård J, et al. Identification of endoribonuclease specific cleavage positions reveals novel targets of RNase III in *Streptococcus pyogenes*. Nucleic Acids Res. 2017;45:2329–2340.

[6] Lyon WR, Gibson CM, Caparon MG. A role for trigger factor and an Rgg-like regulator in the transcription, secretion and processing of the cysteine proteinase of *Streptococcus pyogenes*. EMBO J. 1998;17:6263–6275.

[7] Chaussee MS, Ajdic D, Ferretti JJ. The *rgg* gene of *Streptococcus pyogenes* NZ131 positively influences extracellular SPE B production. Infect Immun. 1999;67:1715–1722.

[8] Anbalagan S, McShan WM, Dunman PM, et al. Identification of Rgg binding sites in the *Streptococcus pyogenes* chromosome. J Bacteriol. 2011;193:4933–4942.

[9] Heath A, DiRita VJ, Barg NL, et al. A two-component regulatory system, CsrR-CsrS, represses expression of three *Streptococcus pyogenes* virulence factors, hyaluronic acid capsule, streptolysin S, and pyrogenic exotoxin B. Infect Immun. 1999;67:5298–5305.

[10] Miller AA, Engleberg NC, DiRita VJ. Repression of virulence genes by phosphorylation-dependent oligomerization of CsrR at target promoters in *S. pyogenes*. Mol Microbiol. 2001;40:976–990.

[11] Graham MR, Smoot LM, Migliaccio CAL, et al. Virulence control in group A Streptococcus by a two-component gene regulatory system: global expression profiling and in vivo infection modeling. Proc Natl Acad Sci USA. 2002;99:13855–13860.

[12] Kietzman CC, Caparon MG. CcpA and LacD.1 affect temporal regulation of *Streptococcus pyogenes* virulence genes. Infect Immun. 2010;78:241–252.

[13] Shelburne SA, Keith D, Horstmann N, et al. A direct link between carbohydrate utilization and virulence in the major human pathogen group A Streptococcus. Proc Natl Acad Sci USA. 2008;105:1698–1703.

[14] Loughman JA, Caparon MG. A novel adaptation of aldolase regulates virulence in *Streptococcus pyogenes*. EMBO J. 2006;25:5414–5422.

[15] Ma Y, Bryant AE, Salmi DB, et al. *vfr*, a novel locus affecting cysteine protease production in *Streptococcus pyogenes*. J Bacteriol. 2009;191:3189–3194.

[16] Shelburne SA, Olsen RJ, Makthal N, et al. An N-Terminal signal peptide of Vfr protein negatively influences RopB-dependent SpeB expression and attenuates virulence in *Streptococcus pyogenes*. Mol Microbiol. 2011;82:1481–1495.

[17] Makthal N, Gavagan M, Do H, et al. Structural and functional analysis of RopB: a major virulence regulator in *Streptococcus pyogenes*. Mol Microbiol. 2016;99:1119–1133.

[18] Kang SO, Caparon MG, Cho KH. Virulence gene regulation by CvfA, a putative RNase: the CvfA-Enolase complex in *Streptococcus pyogenes* links nutritional stress, growth-phase control, and virulence gene expression. Infect Immun. 2010;78:2754–2767.

[19] Chen Z, Mashburn-Warren L, Merritt J, et al. Interference of a *speB* 5' untranslated region partial deletion with mRNA degradation in *Streptococcus pyogenes*. Mol Oral Microbiol. 2017;32:390–403.

[20] Chen Z, Itzek A, Malke H, et al. Multiple roles of RNase Y in *Streptococcus pyogenes* mRNA processing and degradation. J Bacteriol. 2013;195:2585–2594.
